# Subsurface Stabilization of Interstitial Pt Atoms on CeO_2_(111): Rethinking Single‐Atom Catalyst Architectures

**DOI:** 10.1002/anie.202522372

**Published:** 2026-04-20

**Authors:** Shuang Chen, Zairan Yu, Junjun Wang, Jelena Jelic, Wangtao Li, Felix Studt, Yuemin Wang, Christof Wöll

**Affiliations:** ^1^ Institut Für Funktionelle Grenzflächen (IFG) Karlsruher Institut für Technologie (KIT) Eggenstein‐Leopoldshafen Deutschland; ^2^ Engineering Research Center of Environmental Materials and Membrane Technology of Hubei Province School of Materials Science and Engineering Wuhan Institute of Technology Wuhan China; ^3^ Institute of Catalysis Research and Technology (IKFT) Karlsruhe Institute of Technology (KIT) Eggenstein‐Leopoldshafen Germany; ^4^ Institute for Chemical Technology and Polymer Chemistry (ICTP) Karlsruhe Institute of Technology (KIT) Karlsruhe Germany; ^5^ Ordos Laboratory Ordos Inner Mongolia China

**Keywords:** ceria, density functional theory, IRRAS, single‐atom catalysts, surface chemistry

## Abstract

Single‐atom catalysts (SACs) offer maximal efficiency by stabilizing isolated metal atoms on oxidic supports. Platinum on cerium oxide (CeO_2_) is a key SAC system, where adsorbed CO typically exhibits red‐shifted vibrational modes due to Pt back‐donation. Using polarization‐resolved IR spectroscopy on Pt‐deposited CeO_2_(111) single‐crystal surfaces, we do not observe CO vibrational bands below 2140 cm^−1^ at low coverages, indicating that the surface‐bound Pt atoms are not present in detectable amounts. DFT calculations demonstrate that this unexpected observation is consistent with Pt atoms occupying buried interstitial sites. Such subsurface single‐atom sites, not considered in previous studies, are thermodynamically favored at low coverages, adopt an unusual oxidation state, and are inaccessible to direct CO binding. Our findings challenge the prevailing assumption that single atoms remain surface‐bound and highlight the critical role of subsurface interstitial species, prompting a rethinking of how active sites in single‐atom catalysts are stabilized on reducible oxides.

## Graphical abstract

By combining polarization‐resolved IRRAS on atomically flat CeO_2_(111) single crystals with quantitative XPS and DFT calculations, we identify an unusual subsurface interstitial configuration of Pt single atoms that gives rise to a previously unobserved, blue‐shifted CO vibrational mode. The p‐ and s‐polarized IRRAS data further enable precise identification of distinct surface sites on Pt clusters and particles, including terraces, edges, and corners.

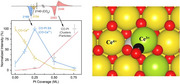

## 1 Introduction

Single‐atom catalysis (SAC) has emerged as a powerful paradigm in heterogeneous catalysis, offering both maximal atom efficiency and access to unique reaction pathways through isolated metal sites [1, 2, 3, 4, 5, 6, 7, 8]. These systems often surpass traditional nanoparticle‐based catalysts in activity, selectivity, and thermal stability, making SAC a promising route toward more efficient and sustainable catalytic technologies.

For certain oxide substrates, such as Fe_3_O_4_, the presence of single, surface‐bound metal atoms has been clearly demonstrated [2]. However, for many other systems, including cerium dioxide (CeO_2_), the nature and stability of isolated metal sites remain under debate. Among these, platinum supported on CeO_2_ (Pt–CeO_2_) stands out due to the strong electronic and structural interplay between Pt atoms and the redox‐active oxide support [9, 10, 11, 12, 13].

Over the past decade, Pt–CeO_2_ single‐atom catalysts have been the focus of intense research, particularly in key reactions such as CO oxidation, the water–gas shift reaction, and selective hydrogenations [14, 15, 16, 17]. However, despite this progress, fundamental questions about structural dynamics and reaction mechanisms at the atomic scale remain unresolved. The identity of the active site at low Pt coverages, in particular, is highly controversial: while some studies attribute the activity to isolated Pt atoms on the CeO_2_ surface [9, 17, 18, 19], others present evidence that contradicts this view [20, 21, 22, 23, 24]. To address these challenges, infrared (IR) spectroscopy using carbon monoxide (CO) as a vibrational probe has proven invaluable, offering detailed insight into the electronic structure and coordination environment of surface species [25, 26, 27, 28, 29, 30, 31]. Among the techniques available to bridge the pressure and materials gaps in surface science, CO‐based IR spectroscopy is especially versatile. Vibrational bands observed under operando conditions in diffuse reflectance infrared Fourier transform spectroscopy (DRIFTS) on powders can be rigorously assigned using Infrared reflection–absorption spectroscopy (IRRAS) data obtained from model substrates and validated by theoretical calculations [32, 33].

The assignment of CO stretching frequencies in Pt–ceria catalysts remains debated, as red‐shifted bands attributed to Pt single atoms often overlap with signals from Pt clusters. Polarization‐resolved IRRAS on oxide single crystals provides the spectral precision needed to benchmark theoretical predictions [33, 34], but its application has been limited by the inherently low reflectivity of dielectric substrates. To date, no such measurements have been reported for Pt on CeO_2_, leaving the vibrational signature of CO bound to isolated Pt atoms experimentally unresolved.

Here we present a comprehensive analysis of CO‐IRRAS data for Pt deposited on CeO_2_(111) single‐crystal surfaces recorded using both p‐ and s‐polarized light. Pt coverages were precisely quantified by quartz crystal microbalance (QCM) and x‐ray photoelectron spectroscopy (XPS), while the fraction of uncovered ceria was tracked via the CO vibrational band characteristic of pristine CeO_2_(111). Particular focus is placed on Pt single atoms, with interpretation of the IRRAS data supported by CO‐SLIR (surface‐ligand IR) measurements [31, 32] and density functional theory (DFT) calculations.

## 2 Results and Discussion

### 2.1 Pt Clusters/Particles Deposited on CeO_2_(111)

**FIGURE 1:**
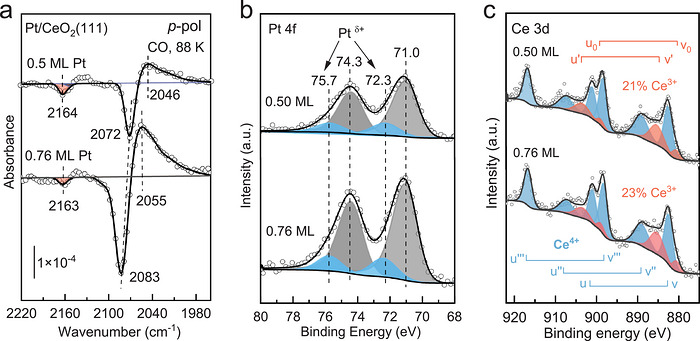
Polarization‐resolved IRRAS and grazing‐emission XPS characterization of Pt clusters/particles deposited on CeO_2_(111) single crystal surfaces. (a) p‐polarized IRRAS data recorded after CO saturation adsorption at 88 K on the Pt‐deposited CeO_2_(111) surfaces at high Pt coverages (0.50 and 0.76 ML); (b) deconvoluted Pt 4f and (c) Ce 3d spectra of 0.50 ML and 0.76 ML Pt/CeO_2_(111).

Figure 1a shows polarization‐resolved IRRAS data collected after CO saturation at 88 K on Pt‐deposited CeO_2_(111) surfaces with high Pt coverages of 0.50 and 0.76 monolayer (ML). Pt/CeO_2_(111) samples were prepared by well‐controlled deposition of Pt at 300 K, followed by annealing in UHV at 700 K for 10 min (for details, see Supporting information). Here, 1 ML of Pt is defined as one Pt atom per surface unit cell of CeO_2_(111), corresponding to 7.9 × 10^14^ atoms/cm^2^. The clean CeO_2_(111) surface exhibits a sharp band at 2154 cm^−1^, blue‐shifted relative to the gas‐phase CO value at 2143 cm^−1^, which is assigned to CO bound to sevenfold‐coordinated surface Ce^4+^ sites (Ce_7c_^4+^) [35]. Following Pt deposition at 0.50 and 0.76 ML, broad red‐shifted CO bands appear at 2100–2000 cm^−1^, assigned to CO bound to Pt clusters or particles, consistent with previous reports. [36, 37, 38, 39] As these Pt‐related IR signals grow, the 2154 cm^−1^ feature of the pristine CeO_2_(111) surface diminishes, indicating progressive Pt cluster formation and surface coverage. XPS analysis (Figure 1b,c) confirms that at 0.75 ML, Pt is predominantly metallic (4f_7/2_/4f_5/2_ doublet at 71.0/74.3 eV), with only a minor contribution from positively charged Pt^δ^^+^ species (72.3/75.7 eV) (Figure 1b).

**FIGURE 2:**
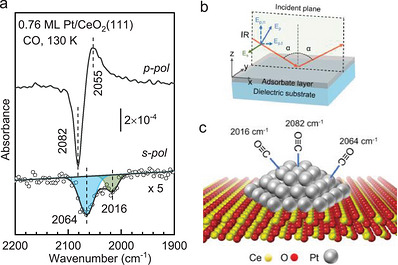
Pt clusters/particles deposited on CeO_2_(111) single‐crystal surfaces characterized by polarization‐resolved IRRAS. (a) p‐ and s‐polarized IRRAS data recorded after CO saturation adsorption at 130 K on the Pt‐deposited CeO_2_(111) surfaces with a high Pt coverage of 0.76 ML. (b) Schematic representation of the interaction between incident IR light and molecular adsorbates on dielectric substrate surfaces. (c) Schematic illustration of distinct CO species adopting site‐specific adsorption geometries: upright adsorption on high‐coordinated terrace Pt sites and tilted adsorption on undercoordinated edge and corner sites.

Interestingly, the p‐polarized IRRAS data show a splitting of the Pt‐related CO bands with an unusual sign reversal (see Figures 1a and 2a). The negative features at 2072–2083 cm^−1^ are due to upright CO on high‐coordinated Pt atop sites on terrace planes, excited by the normal component of the p‐polarized IR radiation (*E*_p_,_n_, see Figure 2b,c). In contrast, the positive bands centered at 2048–2055 cm^−1^, not reported previously [29], are characteristic of tilted CO on undercoordinated Pt atop sites at particle edges and corners, excited by the tangential component (*E*_p_,_t_, see Figure 2b,c). These assignments are further supported by additional IRRA spectra recorded in s‐polarization (see Figure 2a)—an approach not feasible to oxide thin films on metallic substrates due to the surface selection rule [40, 41]. The s‐polarized spectrum reveals two well‐resolved negative bands at 2064 and 2016 cm^−1^, which originate from CO bound to edge and corner Pt atoms, respectively, with the molecular axis oriented predominantly parallel to the substrate and characterized by reduced coordination numbers (Figure 2c).

Temperature‐dependent IRRAS measurements allowed to estimate the binding energy of these red‐shifted CO species, which amount to about 1.55 eV (Supporting Information, Figure S1). The strong interaction between CO and Pt clusters/particles arises from the significant electronic back‐donation from Pt 5d states into the CO 2π* antibonding orbital, in line with the substantial red‐shift of the CO stretching vibrations.

### 2.2 Pt Single Atoms Deposited on CeO_2_(111)

**FIGURE 3:**
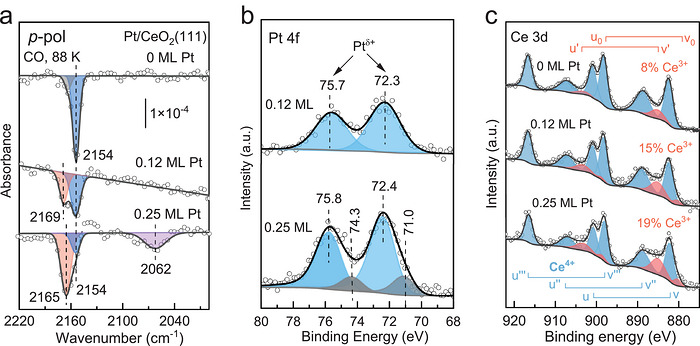
Polarization‐resolved IRRAS and grazing‐emission XPS characterization of Pt single atoms and small clusters deposited on CeO_2_(111) single‐crystal surfaces. (a) p‐polarized IRRAS data recorded after CO saturation adsorption at 88 K on the pristine and Pt‐deposited CeO_2_(111) surfaces at low Pt coverages (0.12 and 0.25 ML); (b) deconvoluted Pt 4f spectra of 0.12 and 0.25 ML Pt/CeO_2_(111); (c) deconvoluted Ce 3d spectra of pristine and Pt (0.12, 0.25 ML) deposited CeO_2_(111).

While the results at higher coverages (>0.5 ML) are consistent with previously reported Pt cluster/particle formation, the low‐coverage regime reveals unexpected behavior. As shown in Figure 3a, the red‐shifted Pt‐related CO bands (2100–2000 cm^−1^) vanish entirely below 0.12 ML, even though QCM and XPS unambiguously confirm the presence of Pt (Figure 3b). The effect of Pt deposition is further demonstrated by attenuation of the pristine 2154 cm^−1^ peak and the emergence of a previously unreported band at 2169 cm^−1^ (Figure 3a). The new 2169 cm^−1^ CO species is only weakly bound (< 0.3 eV from temperature‐dependent IRRAS), in pronounced contrast to CO adsorption on Pt clusters. Importantly, no additional CO bands indicative of surface defect sites are observed. XPS (Figure 3b,c) reveals Pt^d+^ species (d < 2) together with ∼15% Ce^3+^, leading us to hypothesize that, in this low‐coverage regime, isolated Pt atoms do not remain on the surface but migrate into subsurface sites.

Upon increasing the Pt coverage to 0.25 ML (Figure 3a), the 2169 cm^−1^ band shifts slightly to 2165 cm^−1^ and becomes predominant, while a weak red‐shifted, negative CO band appears at 2062 cm^−1^, characteristic of CO bound to low‐coordinated Pt sites in small clusters. The corresponding Pt 4f (Figure 3b) and Ce 3d (Figure 3c) XPS data show that Pt remains predominantly in the Pt^d+^ state, with the fraction of reduced Ce^3+^ species increasing slightly to 19%.

**FIGURE 4:**
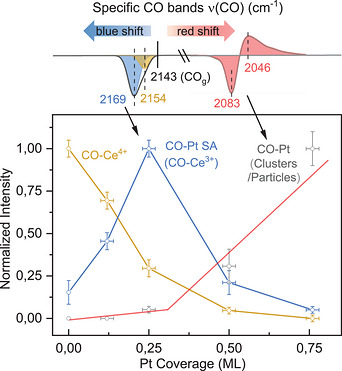
Quantitative analysis of distinct CO vibrations. Coverage‐dependent intensity evolution of specific CO bands characteristic of distinct adsorption sites: Pt single atoms (CO‐Pt SA, ∼2169 cm^−1^); Pt small clusters and particles (CO‐Pt, 2100‐2000 cm^−1^) surface Ce cations (CO‐Ce^4+^, 2154 cm^−1^). The Pt coverage was determined by QCM and XPS (see text).

Figure 4 summarizes the integrated intensity evolution of various CO bands as a function of Pt coverage, determined by QCM. (i) The 2154 cm^−1^ band, reflecting the fraction of the CeO_2_(111) surface not covered by Pt, decreases rapidly with increasing coverage. (ii) CO bands attributed to Pt clusters and particles (2100–2000 cm^−1^) vanish almost completely at coverages below 0.25 ML. (iii) The CO band associated with Pt single atoms at 2169 cm^−1^ reaches its maximum intensity at 0.25 ML and then declines sharply at higher coverages. Spectroscopic analysis indicates the coexistence of Pt single atoms and small Pt clusters at intermediate coverages. Since this blue‐shifted frequency has not been reported previously, the location of the deposited Pt atoms cannot be inferred directly from the band position alone, necessitating a thorough theoretical analysis.

### 2.3 Theoretical Results

Initial calculations for a bare (2 × 2) CeO_2_(111) unit cell show CO binding to Ce_7c_^4+^ sites with  *E*_ads_, CO  =  –0.21 eV and n(CO)  =  2150  cm^−1^ (Figure S2), consistent with previous reports [34]. Geometry optimization of a surface‐bound Pt atom on CeO_2_(111) at 0.25 ML coverage (one Pt per (2 × 2) unit cell) identifies a bridge site above two surface O atoms as the most stable configuration (Figures S2 and S3), also consistent with earlier studies [29]. In this state, Pt carries a formal charge of +1.3 (Table 1) and binds CO with *E*_ads_, CO  =  –2.28 eV and ν(CO)  =  2047 cm^−1^ (Table 2). While this red‐shift matches previous experimental IR data, it does not account for the newly observed blue‐shifted, weakly bound CO species.

**TABLE 1:** Calculated stabilities and Pt oxidation state of different Pt/CeO_2_(111) structures relative to Pt_bulk_ and CeO_2_(111) in eV.

	Pt coverage	∆*E*	Pt oxidation state^a^
Pt@CeO_2_(111) in subsurface	1/4 ML	+ 1.61	+ 1.9
Pt@CeO_2_(111) in subsurface	1/9 ML	+ 1.23	+ 1.9
Pt@CeO_2_(111) on top surface	1/4 ML	+ 1.84	+ 1.3
Pt@CeO_2_(111) on top surface	1/9 ML	+ 1.77	+ 1.3

^a^ Derived from Bader charge analysis (Figure S4 and Table S1a,b).

**TABLE 2:** Calculated CO binding energies (in eV), vibrational frequencies (scaled and in cm^−1^), and vibration intensities for Pt/CeO_2_(111) with Pt single atoms on the surface and in subsurface interstitial sites. CO has been scaled with a factor of 1.008.

	∆*H*_CO_	Vibration frequency (cm^−1^)	Intensity ((D/Å)^2^/amu)
1/4 ML Pt—in subsurface	−0.30	2156	3.6426
1/4 ML Pt—on top surface	−2.28	2047	32.6469
1/4 ML Pt—on top surface^a^	−1.46^b^	2120/2050^c^	19.6347/0.0764^d^
1/9 ML Pt—in subsurface	−0.29	2174	3.9740
1/9 ML Pt—on top surface	−2.35	2031	38.0939
1/9 ML Pt—on top surface^a^	−1.29^b^	2118/2067^c^	24.2305/0.0007^d^

^a^ Adsorption of 2 CO molecules

^b^ Differential heat of adsorption of second CO molecule

^c^ Symmetric and asymmetric CO vibrations

^d^ Intensities of symmetric and asymmetric CO vibration modes.

To resolve this discrepancy with the experimental data, subsurface Pt configurations were explored. Since in earlier work [20] we reported that replacing Ce atoms in the ceria bulk with Pt carries a high energy penalty (1.62 eV, see also Refs [11, 42, 43].), we focused on interstitial rather than substitutional sites. Although such configurations have rarely been considered in previous theoretical work, we found—surprisingly—that a Pt atom occupying the empty space between O^2−^ ions in the bulk interstitial is energetically more favorable by –0.23 eV (see Table 1) than on‐surface sites. Using a larger (3×3) unit cell (1/9 ML coverage), representative of the experimental regime (0.12 ML), this stabilization increases to –0.54 eV, with Pt adopting a formal charge of +1.9 (Figure S4 and Table S1a,b).

**FIGURE 5:**
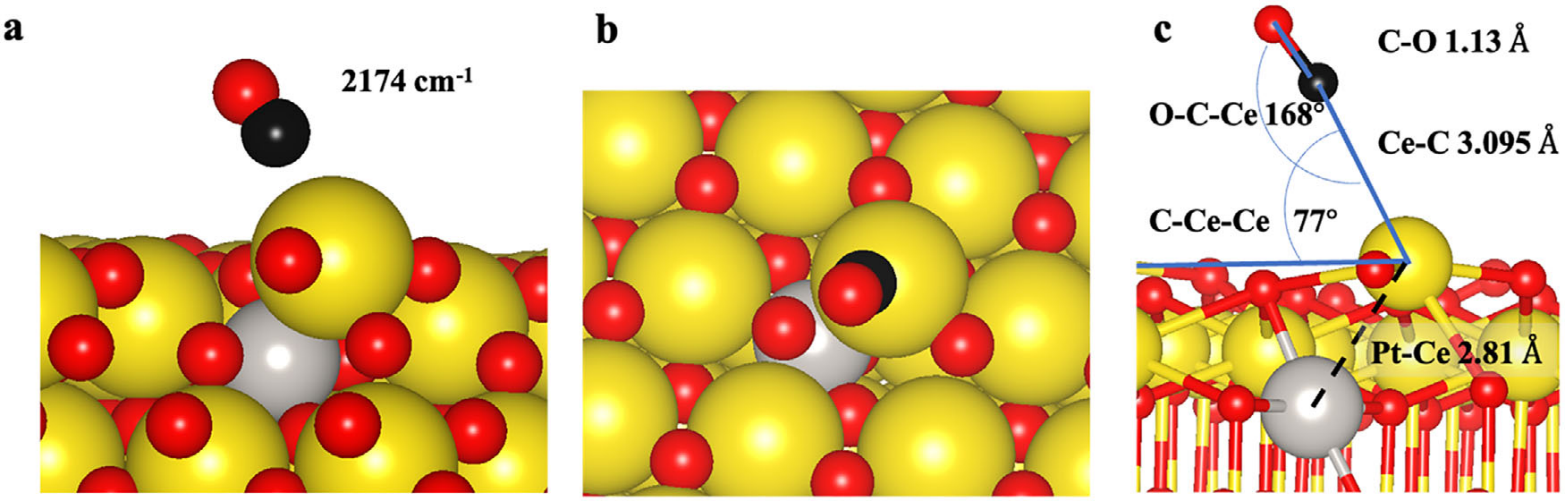
DFT‐optimized structure of single‐atom Pt/CeO_2_(111) model catalyst. (a) Side view, (b) top view, and (c) specific bond distances and angles of the optimized geometry of CO on Ce atoms on top interstitial Pt stabilized in the subsurface of CeO_2_(111).

In this unusual interstitial geometry (see Figure 5), the Pt atom is buried beneath the CeO_2_(111) surface in a fourfold coordination with surrounding O^2−^ ions, rendering it inaccessible to CO. As a consequence, a nearby surface Ce^4+^ cation is displaced upwards by 1.3 Å (see Figure 5c), becoming reduced to Ce^3+^ together with an adjacent Ce atom (Figure S5). This redox process facilitates the oxidation of Pt^0^ to Pt^1.8+^. CO binds to the protruding Ce^3+^ site with a slightly tilted geometry (74°), showing an adsorption energy of *E*_ads_, CO  =  –0.29 eV and a vibrational frequency of n(CO)  =  2174 cm^−1^ (Table 2), closely matching our experimental data. Notably, the calculated oxidation state of subsurface Pt aligns well with XPS measurements, while surface‐bound Pt remains less oxidized. Furthermore, we find that CO adsorption on distinct positively charged surface Pt single‐atom sites at coverages of 0.12 and 0.25 ML—including configurations with one or two CO molecules per Pt site—leads to a red shift in the calculated stretching frequencies, in marked contrast to our experimental observations (see Table 2).

The 0.38 eV difference in subsurface interstitial Pt stability between 1/4 and 1/9 ML (Table 1) arises from strain and relaxation. In the smaller (2 × 2) cell, overlapping displacement fields hinder subsurface insertion, whereas the larger (3×3) cell better accommodates lattice distortions, stabilizing subsurface Pt at low coverage.

To assess the thermal stability of Pt single atoms on CeO_2_, it is essential to compare their energies to bulk Pt as a reference state (see Table 1). On this basis, all configurations studied—both surface and subsurface—are thermodynamically unstable with respect to bulk Pt. However, subsurface Pt is consistently more stable than surface‐bound Pt atoms, and this relative stability increases further at lower coverages.

The computed activation barrier for Pt diffusion into the subsurface interstitial site (2.03 eV, see Supporting Information, Figures S6 and S7) is consistent with the mild 700 K annealing applied after deposition, comparable to temperatures used in ceria‐based catalysts. Assuming a standard attempt frequency of 10^13^ s^−1^, Pt incorporation at 700 K is expected to occur within 1 min, well below the experimental annealing time.

## 3 Conclusion

Our combined IR spectroscopic and DFT study reveals that, contrary to prevailing assumptions, single Pt atoms deposited on CeO_2_(111) surfaces are not present in detectable amounts at the surface at low coverages, consistent with migration into subsurface sites. This previously unrecognized configuration gives rise to a distinct, blue‐shifted CO vibrational band, reflecting a weakly bound CO species and minimal back‐donation from the embedded Pt atom. Thermodynamically, buried interstitial Pt is predicted to be more stable than surface‐bound Pt, particularly at low coverages, a trend supported by both experiment and theory. These findings redefine the structural landscape of single‐atom catalysts on reducible oxides and underscore the important role of considering subsurface incorporation, in particular at interstitial sites, as an intrinsic design parameter. Given the generality of strain, redox, and coordination effects across oxide supports, similar subsurface interstitial stabilization may occur for other metal‐oxide combinations [44], suggesting a broader need to reassess surface‐bound assumptions in the design of the next‐generation SACs. The catalytic activity of such subsurface interstitial sites will be addressed in future work on powder samples, as direct catalytic measurements are not feasible for single‐crystal model systems.

## Conflicts of Interest

The authors declare no conflicts of interest.

## Supporting information




**Supporting File**: anie72230‐sup‐0001‐SuppMat.pdf.

## Data Availability

The data that support the findings of this study are available in the Supporting information of this article.

## References

[1] B. Qiao , A. Wang , X. Yang , et al., “Single‐atom Catalysis of CO Oxidation Using Pt1/FeOx,” Nature Chemistry 3, no. 8 (2011): 634–641, 10.1038/nchem.1095. 21778984

[2] J. Hulva , M. Meier , R. Bliem , et al., “Unraveling CO Adsorption on Model Single‐atom Catalysts,” Science 371, no. 6527 (2021): 375–379, 10.1126/science.abe5757. 33479148

[3] X. X. Shi , Z. L. Wen , Q. Q. Gu , et al., “Metal–Support Frontier Orbital Interactions in Single‐Atom Catalysis,” Nature 640 (2025): 668–675, 10.1038/s41586-025-08747-z. 40175541

[4] Y. Zhai , D. Pierre , R. Si , et al., “Alkali‐stabilized Pt‐OHx Species Catalyze Low‐temperature Water‐gas Shift Reactions,” Science 329, no. 5999 (2010): 1633–1636, 10.1126/science.1192449. 20929844

[5] L. Liu , U. Díaz , R. Arenal , G. Agostini , P. Concepción , and A. Corma , “Generation of Subnanometric Platinum With High Stability During Transformation of a 2D Zeolite Into 3D,” Nature Materials 16, no. 1 (2017): 132–138, 10.1038/nmat4757. 27669051

[6] X. Cui , W. Li , P. Ryabchuk , K. Junge , and M. Beller , “Bridging Homogeneous and Heterogeneous Catalysis by Heterogeneous Single‐Metal‐Site Catalysts,” Nature Catalysis 1, no. 6 (2018): 385–397, 10.1038/s41929-018-0090-9.

[7] V. Muravev , G. Spezzati , Y.‐Q. Su , et al., “Interface dynamics of Pd–CeO_2_ Single‐Atom Catalysts During CO oxidation,” Nature Catalysis 4, no. 6 (2021): 469–478, 10.1038/s41929-021-00621-1.

[8] L. Kang , B. Zhu , Q. Gu , et al., “Light‐Driven Propane Dehydrogenation by a Single‐atom Catalyst Under near‐ambient Conditions,” Nature Chemistry 17 (2025): 890–896, 10.1038/s41557-025-01766-3. 40119166

[9] X. Li , X. I. Pereira‐Hernández , Y. Chen , et al., “Functional CeOx Nanoglues for Robust Atomically Dispersed Catalysts,” Nature 611, no. 7935 (2022): 284–288, 10.1038/s41586-022-05251-6. 36289341

[10] J. Jones , H. Xiong , A. T. DeLaRiva , et al., “Thermally Stable Single‐atom Platinum‐on‐Ceria Catalysts via Atom Trapping,” Science 353, no. 6295 (2016): 150–154, 10.1126/science.aaf8800. 27387946

[11] N. Daelman , M. Capdevila‐Cortada , and N. López , “Dynamic Charge and Oxidation state of Pt/CeO_2_ Single‐atom Catalysts,” Nature Materials 18, no. 11 (2019): 1215–1221, 10.1038/s41563-019-0444-y. 31384029

[12] Q. Fu , H. Saltsburg , and M. Flytzani‐Stephanopoulos , “Active Nonmetallic Au and Pt Species on Ceria‐Based Water‐gas Shift Catalysts,” Science 301, no. 5635 (2003): 935–938, 10.1126/science.1085721. 12843399

[13] A. Trovarelli and J. Llorca , “Ceria Catalysts at Nanoscale: How Do Crystal Shapes Shape Catalysis?,” ACS Catalysis 7, no. 7 (2017): 4716–4735, 10.1021/acscatal.7b01246.

[14] K. Ding , A. Gulec , A. M. Johnson , et al., “Identification of Active Sites in CO Oxidation and Water‐Gas Shift Over Supported Pt Catalysts,” Science 350, no. 6257 (2015): 189–192, 10.1126/science.aac6368. 26338796

[15] A. Wang , J. Li , and T. Zhang , “Heterogeneous Single‐atom Catalysis,” Nature Reviews Chemistry 2, no. 6 (2018): 65–81, 10.1038/s41570-018-0010-1.

[16] A. M. Gänzler , M. Casapu , P. Vernoux , et al., “Tuning the Structure of Platinum Particles on Ceria In Situ for Enhancing the Catalytic Performance of Exhaust Gas Catalysts,” Angewandte Chemie International Edition 56, no. 42 (2017): 13078–13082, 10.1002/anie.201707842. 28796399

[17] L. Nie , D. Mei , H. Xiong , et al., “Activation of Surface Lattice Oxygen in Single‐atom Pt/CeO_2_ for Low‐temperature CO Oxidation,” Science 358, no. 6369 (2017): 1419–1423, 10.1126/science.aao2109. 29242344

[18] Z. Zhang , J. Tian , Y. Lu , et al., “Memory‐Dictated Dynamics of Single‐atom Pt on CeO_2_ for CO Oxidation,” Nature Communications 14, no. 1 (2023): 2664, 10.1038/s41467-023-37776-3. PMC10169862 37160890

[19] D. Jiang , Y. Yao , T. Li , et al., “Tailoring the Local Environment of Platinum in Single‐Atom Pt 1 /CeO_2_ Catalysts for Robust Low‐Temperature CO Oxidation,” Angewandte Chemie International Edition 133, no. 50 (2021): 26258–26266, 10.1002/ange.202108585. 34346155

[20] F. Maurer , J. Jelic , J. Wang , et al., “Tracking the Formation, Fate and Consequence for Catalytic Activity of Pt Single Sites on CeO_2_ ,” Nature Catalysis 3, no. 10 (2020): 824–833, 10.1038/s41929-020-00508-7.

[21] H. Jeong , O. Kwon , B.‐S. Kim , et al., “Highly Durable Metal Ensemble Catalysts With Full Dispersion for Automotive Applications Beyond Single‐atom Catalysts,” Nature Catalysis 3, no. 4 (2020): 368–375, 10.1038/s41929-020-0427-z.

[22] H. Wang , J.‐X. Liu , L. F. Allard , et al., “Surpassing the Single‐Atom Catalytic Activity Limit Through Paired Pt‐O‐Pt Ensemble Built From Isolated Pt1 Atoms,” Nature Communications 10, no. 1 (2019): 3808, 10.1038/s41467-019-11856-9. PMC6707320 31444350

[23] B. B. Sarma , F. Maurer , D. E. Doronkin , and J.‐D. Grunwaldt , “Design of Single‐atom Catalysts and Tracking Their Fate Using Operando and Advanced X‐ray Spectroscopic Tools,” Chemical Review 123, no. 1 (2022): 379–444, 10.1021/acs.chemrev.2c00495. PMC9837826 36418229

[24] B. Bohigues , S. Rojas‐Buzo , D. Salusso , et al., “Overcoming Activity/Stability Tradeoffs in CO Oxidation Catalysis by Pt/CeO_2_ ,” Nature Communications 16, no. 1 (2025): 7451, 10.1038/s41467-025-62726-6. PMC12344003 40796774

[25] X. I. Pereira‐Hernández , A. DeLaRiva , V. Muravev , et al., “Tuning Pt‐CeO_2_ Interactions by High‐temperature Vapor‐phase Synthesis for Improved Reducibility of Lattice Oxygen,” Nature Communications 10, no. 1 (2019): 1358, 10.1038/s41467-019-09308-5. PMC6433950 30911011

[26] M. Kottwitz , Y. Li , R. M. Palomino , et al., “Local Structure and Electronic state of Atomically Dispersed Pt Supported on Nanosized CeO_2_ ,” ACS Catalysis 9, no. 9 (2019): 8738–8748, 10.1021/acscatal.9b02083.

[27] X. Tang , S. Ge , Y. Lv , et al., “Blocking the Operando Formation of Single‐Atom Spectators by Interfacial Engineering,” Angewandte Chemie International Edition 64 (2025): e202505507, 10.1002/anie.202505507. 40178203

[28] H. Lei , N. Zhang , S. Hu , et al., “Thermally Triggered Redox Flexibility of Pt/CeO_2_ Cluster Catalyst Against In‐Situ Atomic Redispersion,” Angewandte Chemie International Edition 64 (2025): e202509239, 10.1002/anie.202509239. 40433896

[29] W. Wan , J. Geiger , N. Berdunov , et al., “Highly Stable and Reactive Platinum Single Atoms on Oxygen Plasma‐Functionalized CeO_2_ Surfaces: Nanostructuring and Peroxo Effects,” Angewandte Chemie International Edition 61, no. 20 (2022): e202112640, 10.1002/anie.202112640. 35243735 PMC9315031

[30] D. Zhou , J. Wang , M. Jian , et al., “Fine‐tuned Coordination Environment of Pt‐Fe‐Pt Active Site for Selective Heterogeneous Hydrogenation of Crotonaldehyde,” Chemistry 11, no. 5 (2025): 102380, 10.1016/j.chempr.2024.11.018.

[31] C. Wöll , “Structure and Chemical Properties of Oxide Nanoparticles Determined by Surface‐ligand IR Spectroscopy,” ACS Catalysis 10, no. 1 (2019): 168–176, 10.1021/acscatal.9b04016.

[32] C. Yang , H. Idriss , Y. Wang , and C. Wöll , “Surface Structure and Chemistry of CeO_2_ Powder Catalysts Determined by Surface‐ligand Infrared Spectroscopy (SLIR),” Accounts of Chemical Research 57, no. 22 (2024): 3316–3326, 10.1021/acs.accounts.4c00529. 39476853 PMC11580167

[33] P. G. Lustemberg , C. Yang , Y. Wang , C. Wöll , and M. V. Ganduglia‐Pirovano , “Vibrational Frequencies of CO Bound to all Three Low‐index Cerium Oxide Surfaces: A Consistent Theoretical Description of Vacancy‐induced Changes Using Density Functional Theory,” Journal of Chemical Physics 159, no. 3 (2023): 034704, 10.1063/5.0153745. 37462286

[34] P. G. Lustemberg , P. N. Plessow , Y. Wang , et al., “Vibrational Frequencies of Cerium‐oxide‐bound CO: A Challenge for Conventional DFT Methods,” Physical Review Letter 125, no. 25 (2020): 256101, 10.1103/PhysRevLett.125.256101. 33416353

[35] C. Yang , X. Yu , S. Heißler , et al., “Surface Faceting and Reconstruction of Ceria Nanoparticles,” Angewandte Chemie International Edition 56 (2017): 375–379, 10.1002/anie.201609179. 27925439

[36] C. Klünker , M. Balden , S. Lehwald , and W. Daum , “CO Stretching Vibrations on Pt (111) and Pt (110) Studied by Sumfrequency Generation,” Surface Science 360, no. 1‐3 (1996): 104–111.

[37] A. Bourane , “Heats of Adsorption of the Linear CO Species on Pt/Al_2_O_3_ Using Infrared Spectroscopy: Impact of the Pt Dispersion,” Journal of Catalysis 218, no. 2 (2003): 447–452, 10.1016/S0021-9517(02)00183-5.

[38] J. Wang , E. Sauter , A. Nefedov , et al., “Dynamic Structural Evolution of Ceria‐Supported Pt Particles: A Thorough Spectroscopic Study,” Journal of Physical Chemistry C 126 (2022): 9051–9058, 10.1021/acs.jpcc.2c02420.

[39] H. A. Aleksandrov , K. M. Neyman , K. I. Hadjiivanov , and G. N. Vayssilov , “Can the State of Platinum Species be Unambiguously Determined by the Stretching Frequency of an Adsorbed CO Probe Molecule?,” Physical Chemistry Chemical Physics 18, no. 32 (2016): 22108–22121, 10.1039/C6CP03988J. 27444400

[40] C. Yang and C. Wöll , “Infrared Reflection‐Absorption Spectroscopy (IRRAS) Applied to Oxides: Ceria as a Case Study,” Surface Science 749 (2024): 122550, 10.1016/j.susc.2024.122550.

[41] Y. Wang and C. Wöll , “IR Spectroscopic Investigations of Chemical and Photochemical Reactions on Metal Oxides: Bridging the Materials Gap,” Chemical Society Reviews 46, no. 7 (2017): 1875–1932, 10.1039/C6CS00914J. 28221385

[42] Y. Q. Su , Y. F. Wang , J. X. Liu , et al., “Theoretical Approach to Predict the Stability of Supported Single‐Atom Catalysts,” ACS Catalysis 9, no. 4 (2019): 3289–3297, 10.1021/acscatal.9b00252.

[43] D. Kunwar , S. L. Zhou , A. DeLaRiva , et al., “Stabilizing High Metal Loadings of Thermally Stable Platinum Single Atoms on an Industrial Catalyst Support,” ACS Catalysis 9, no. 5 (2019): 3978–3990, 10.1021/acscatal.8b04885.

[44] H. Qiu , F. Gallino , C. Di Valentin , and Y. Wang , “Shallow Donor States Induced by In‐Diffused Cu in ZnO: A Combined HREELS and Hybrid DFT Study,” Physical Review Letter 106, no. 6 (2011): 066401, 10.1103/PhysRevLett.106.066401. 21405480

