# Supplementary material for: Subsurface Stabilization of Interstitial Pt Atoms on CeO2(111): Rethinking Single‐Atom Catalyst Architectures
Source: Angew Chem Int Ed Engl. 2026 Apr 20;65(25):e22372. doi: 10.1002/anie.202522372 (PMC13266957; doi:10.1002/anie.202522372)
Supplement: Supplementary file 1 — Supporting File: anie72230‐sup‐0001‐SuppMat.pdf. [file ANIE-65-e22372-s001.pdf]

Supplementary Information

Subsurface Stabilization of Interstitial Pt Atoms on CeO_2(111): Rethinking Single-Atom Catalyst Architectures

Shuang Chen,[1†] Zairan Yu,[1†] Junjun Wang,[2†] Jelena Jelic,[3†] Wangtao Li,[1] Felix Studt,[3,4*]
Yuemin Wang,[1*] Christof Wöll[1*]

[1]Institute of Functional Interfaces (IFG), Karlsruhe Institute of Technology (KIT), 76344 Eggenstein-Leopoldshafen, Germany.

[2]Engineering Research Center of Environmental Materials and Membrane Technology of Hubei Province, School of Materials Science and Engineering, Wuhan Institute of Technology, Wuhan 430074, China.

[3]Institute of Catalysis Research and Technology (IKFT), Karlsruhe Institute of Technology (KIT), 76344 Eggenstein-Leopoldshafen, Germany.

[4]Institute for Chemical Technology and Polymer Chemistry (ICTP), Karlsruhe Institute of Technology (KIT), 76131 Karlsruhe, Germany.

†These authors contributed equally to this work.

*Corresponding authors: felix-studt@kit.edu (F.S.); yuemin.wang@kit.edu (Y.W.); christof.woell@kit.edu (C.W.)

This file includes:

Supplementary Methods

Supplementary Figures

Supplementary Tables

Supplementary References

Coordinates of all DFT structures

Supplementary Methods

IRRAS and XPS experiments

All experiments were carried out in an advanced ultrahigh vacuum (UHV) system (Prevac, Poland), comprising multiple interconnected chambers equipped with FTIR (Bruker Vertex 80v), XPS, and preparation chambers featuring an Ar^+ sputter gun and an electron beam evaporator (EBV 40A1, Prevac). The system is dedicated to high-sensitivity IR spectroscopic measurements and enables both grazing-incidence IRRAS on oxide single crystals and transmission IR spectroscopy on oxide powders,[1-3] with a reliable absorbance detection limit of ~5×10^{-6} ($\Delta R/R$).

The $CeO_2(111)$ single crystal (5 × 5 × 1 mm, SurfaceNet) was prepared by repeated cycles of Ar^+ sputtering (1 keV, 6 mA, 1×10^{-5} mbar) and annealing at 800 K for 15 min in 1×10^{-5} mbar O_2, resulting in a fully oxidized surface. The temperature was monitored with a type K (NiAl–NiCr) thermocouple attached to the single crystal edge. Surface cleanliness and oxidation states were verified by grazing-emission XPS (VG Scienta R4000 analyzer). Low energy electron diffraction (LEED) (OCI Vacuum Microengineering BDL800) was used to determine the surface structure of ceria (100) single crystals before IRRAS measurements.

The metal platinum (Pt) was deposited onto the prepared pure $CeO_2(111)$ single crystal surface in the preparation UHV chamber using an electron beam evaporator (EBV 40A1, Prevac) at room temperature. Pt coverage on the $CeO_2(111)$ surface was determined using a quartz crystal microbalance (QCM). Prior to deposition, the evaporator was thoroughly degassed to ensure purity. In this study, 1 monolayer (ML) of Pt is defined as one Pt atom per $CeO_2(111)$ surface unit cell, with the area of a $CeO_2(111)$ unit cell being 12.67 Å^2 (1 ML Pt ≈ 7.9×10^{14} atoms/cm^2).[4] The Pt thickness, measured by QCM, was converted to surface coverage θ (ML) on $CeO_2(111)$ using the equation:

$$\theta = \frac{t \cdot \rho \cdot N_A}{M} \cdot \frac{1}{7.9 \times 10^{14}}$$

where θ represents the surface coverage of Pt, t (Å) is the thickness of the deposited Pt on $CeO_2(111)$, ρ is the density of Pt (21.45 g/cm^3 at 20 °C), N_A is Avogadro's constant (6.02 × 10^{23} mol^{-1}), and M is the molar mass of platinum (195.08 g/mol). In addition, the Pt loading was further quantified by XPS analysis of the Pt 4f signal intensity.

Polarization-resolved IRRAS measurements were performed in a separate UHV chamber using both s- and p-polarized light at a fixed grazing incidence angle of 80°. According to the metal oxide surface selection rule, both polarization states must be considered on a dielectric substrate. Under s-polarization, only vibrational modes with transition dipole moments (TDMs) parallel to the surface and perpendicular to the plane of incidence are active, typically observed as negative bands. For p-polarization, the electric field contains both normal ($E_{p,n}$) and tangential ($E_{p,t}$) components: TDMs perpendicular to the surface couple with $E_{p,n}$ (yielding negative bands), whereas TDMs parallel to the surface may interact with $E_{p,t}$ (producing positive bands) or with the s-polarized field, depending on their azimuthal orientation. [5,6]

Prior to the low-temperature CO-IRRAS measurements, $Pt/CeO_2(111)$ samples were annealed in UHV at 700 K for 10 min. Carbon monoxide (99.97%) was introduced into the IR chamber by backfilling through a leak-valve-regulated directional doser connected to a 2 mm diameter tube, positioned 3 cm from the sample surface and 50 cm from the hot-cathode ionization gauge. Under these dosing conditions, the local pressure at the sample surface was approximately two orders of magnitude higher than the chamber pressure indicated by the gauge. During IR data acquisition, the base pressure was maintained below 8 × 10^{-11} mbar, and the sample temperature was controlled between ~80 K (liquid helium cooling) and 1000 K (electron bombardment heating). Before each CO exposure, a background spectrum of the clean sample surface was recorded. All IR spectra were collected by averaging 1024 scans at a spectral resolution of 4 cm^{-1}. CO exposures are reported in units of Langmuir (L), where 1 L corresponds to 1.33 × 10^{-6} mbar·s.

Computational details

Vienna Ab Initio Simulation Package (VASP) [7,8] was used to model different Pt single atom systems on the $CeO_2(111)$ surface. We used a plane-wave basis set with a cutoff energy of 450 eV, the Projector Augmented Wave (PAW) [9,10] method, and the Bayesian Error Estimation Functional with van der Waals correlations (BEEF-vdW) [11,12] exchange correlation functional. To better describe the delocalized Ce f orbitals, we applied GGA+U (U = 5.0 eV) method [13]. We used 2x2 and 3x3 large cells of $CeO_2(111)$ to model different Pt coverages. The infinite slab models are separated by more than 15 Å of vacuum in the z-direction. The atoms in the two uppermost layers were allowed to relax during the geometry optimization. The Brillouin zones were sampled using (4x4x1) and (2x2x1) Monkhorst–Pack k-point grids [14] for 2x2 and 3x3 large unit cells, respectively. The convergence criterion for the geometry optimizations was a maximum force of 0.01 eV/Å. The diffusion barrier search was performed using fixed bond distance constrained. Spin polarization was taken into account in all calculations. The vibrational analyses were performed in the harmonic approximation using the finite difference method with displacements of 0.01 Å.

Supplementary Figures

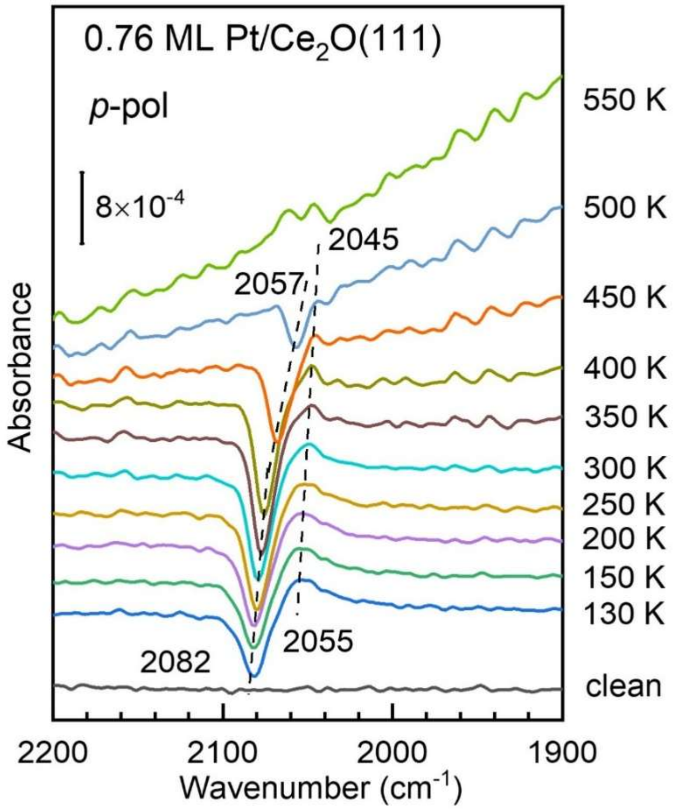

Figure S1. Binding energy of adsorbed CO species determined by temperature-dependent IRRAS data. p-polarized IRRAS data recorded after CO saturation adsorption at 130 K on the Pt deposited CeO_2(111) surfaces with a high Pt coverage of 0.76 ML, subsequently annealing to indicated temperatures.

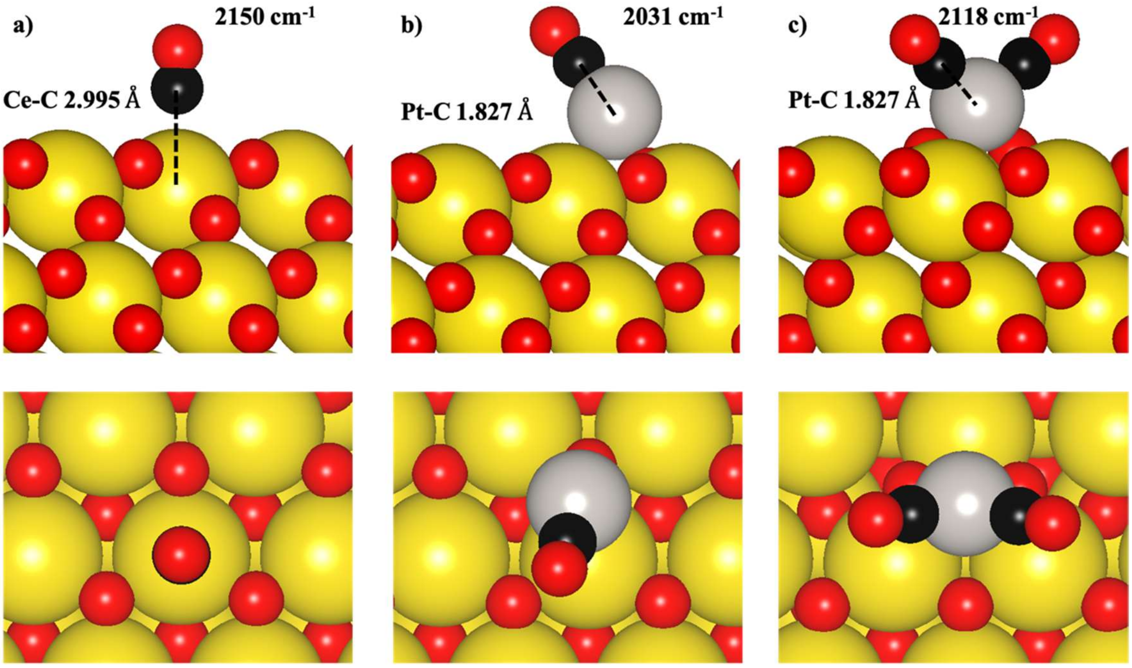

Figure S2. Side and top view of DFT-optimized geometries of: a) CO adsorbed on CeO_2(111) (3x3), b) CO adsorbed on 1/9 ML Pt adatom on CeO_2(111) and c) two CO molecules adsorbed on 1/9 ML Pt adatom on CeO_2(111). Color code: Ce – yellow, O - red, C - black, Pt - gray.

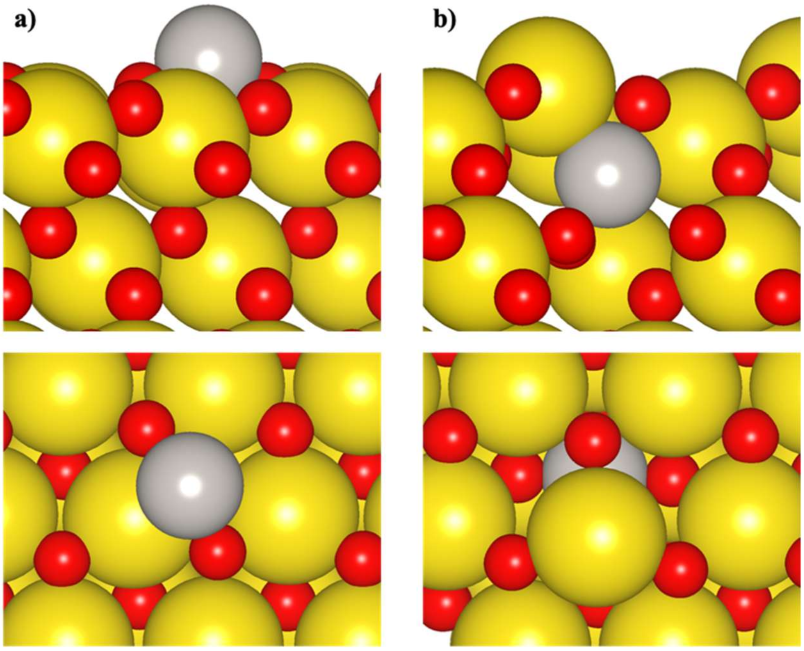

Figure S3. Side and top view of DFT-optimized geometries of: a) 1/4 ML Pt@CeO_2(111) ontop surface and b) 1/4 ML Pt@CeO_2(111) in subsurface. Color code: Ce – yellow, O - red, Pt - gray.

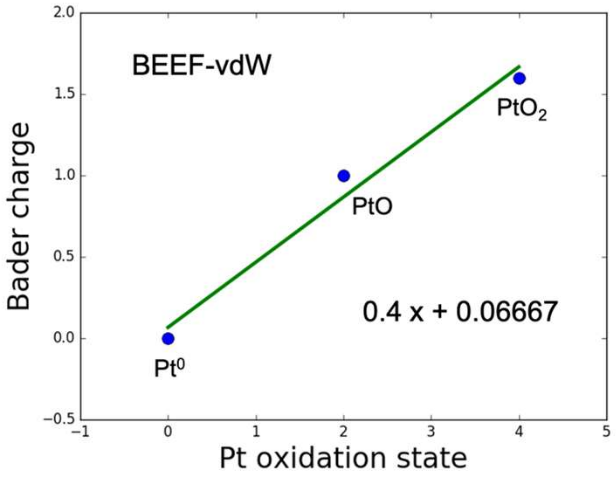

Figure S4. Calculated Bader charge vs the oxidation state of Pt in the metallic Pt^0, PtO and PtO_2**.**

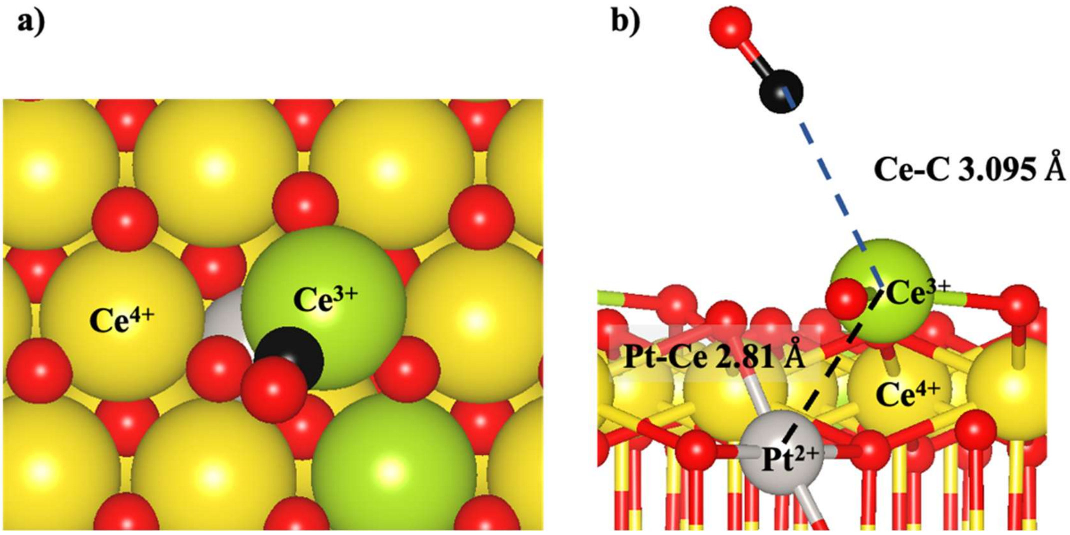

Figure S5. Top and side view of the optimized geometry of CO on interstitial Pt in the subsurface of CeO_2(111). Surface cerium atoms reduced to the +3 oxidation state due to the presence of Pt are shown in green. Color code: Ce^{4+} – yellow, Ce^{3+} – green, O - red, C - black, Pt - gray.

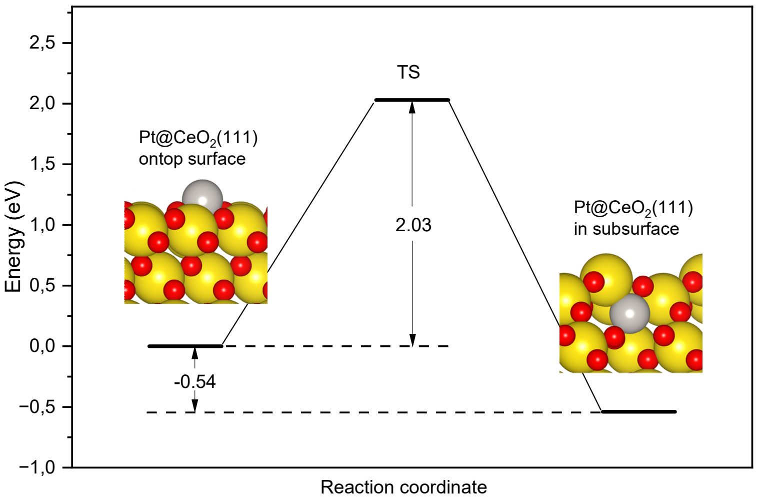

Figure S6. Potential-energy diagram of Pt diffusion from the atop surface to subsurface interstitial sites of $CeO_2(111)$.

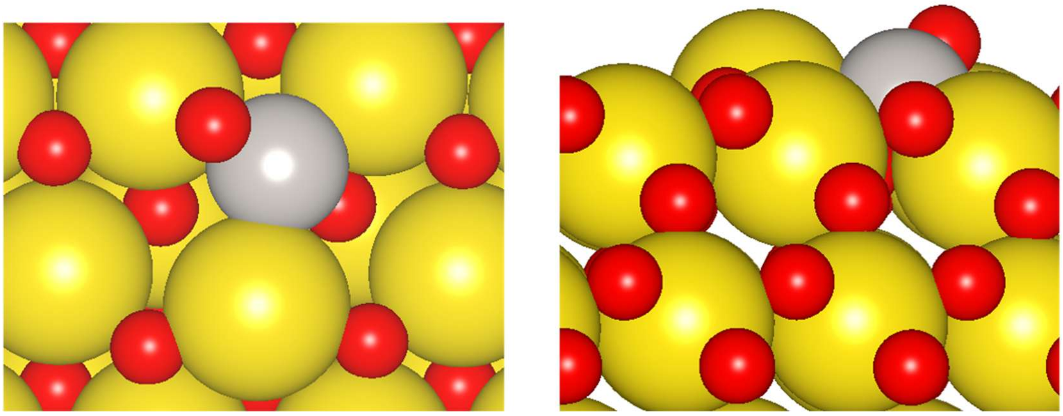

Figure S7. Top and side view of the transition state geometry of Pt diffusion from the surface to the subsurface of $CeO_2(111)$. Color code: Ce – yellow, O - red, Pt - gray.

In the transition states, the heights of the Pt atom and the shifting Ce atom are approximately equal. A new Pt-O bond forms with subsurface oxygen (2.7 Å in the transition state), causing the Pt atom to transition from twofold coordination at the surface to threefold coordination in the inserted state. Further reduction of the Pt height and the formation of the final fourfold-coordinated Pt atom occur with a significantly lower energy barrier.

Supplementary Tables

Table S1a. Calculated Bader charges of Pt, PtO and PtO_2 in the bulk.

	Pt bulk	PtO bulk	PtO_2 bulk
Bader charge	0	1	1.6

Table S1b. Calculated Bader charges of different Pt/CeO_2(111) structures.

	Pt@CeO_2(111) in surface (1/4 ML)	Pt@CeO_2(111) in surface (1/9 ML)	Pt@CeO_2(111) ontop surface (1/4 ML)	Pt@CeO_2(111) ontop surface (1/9 ML)
Bader charge	0.82	0.83	0.61	0.59

Table S2: Calculated CO binding energies (in eV), vibrational frequencies (scaled and in cm-1) for CeO_2(111), Pt/CeO_2(111), and Pt surfaces. CO was scaled by a factor of 1.008.

	ΔH_{CO}	Vibration Frequency(cm^{-1})	Intensity ($(D/Å)^2$ /amu)
CeO_2(111) (2x2)	-0.21	2146	5.6807
CeO_2(111) (3x3)	-0.22	2149	5.4821
1/4 ML Pt – in subsurface	-0.30	2156	3.6426
1/4 ML Pt – ontop surface	-2.38	2047	32.6469
1/4 ML Pt – ontop surface[1]	-1.46[2]	2120/2050[3]	19.6347/0.0764[4]
1/9 ML Pt – in subsurface[5]	-0.29	2174	3.9740
1/9 ML Pt – ontop surface	-2.35	2031	38.0939
1/9 ML Pt – ontop surface[1]	-1.29[2]	2118/2067[3]	24.2305/0.0007[4]
Pt(111) 1/4 ML CO	-1.44	2076	9.9533
Pt(111) 1/2 ML CO	-1.32	2095	6.1209
Pt(211) 1/2 ML CO	-1.78	2063	16.3221
Pt(211) 1 ML CO	-1.42	2072	18.3647

[1] Adsorption of 2 CO molecules, [2] differential heat of adsorption of second CO molecule, [3] symmetric and asymmetric CO vibrations and [4] intensities of symmetric and asymmetric CO vibration modes. [5] Ce^{3+} atoms are atoms number 20 and 24, structure file provided in the appendix.

Table S3. DFT calculated total energies and vibrational frequencies for all calculated structures.

	Total energy (eV)	Vibration Frequency (cm^{-1})
CO(g)	-12.07474	2125.7
Pt(bulk)	-3.1463	/
CeO_2(bulk)	-18.1592	/
CeO_2(111) (2x2)	-285.5974	/
CeO_2(111) (3x3)	-479.1151	/
1/4 ML Pt – ontop surface	-286.9006	/
1/9 ML Pt – ontop surface	-480.4925	/
1/4 ML Pt – in subsurface	-287.1332	/
1/9 ML Pt – in subsurface	-481.0304	/
1/9 ML Pt – substituted	-462.7633	/
Pt(111)	-44.6841	/
Pt(211)	-66.1781	/
CO/CeO_2(111) (2x2)	-297.8830	27.7i, 66.9, 88.3, 183.2, 215.2, 2129.0
CO/CeO_2(111) (3x3)	-491.411	51.5i, 39.2, 94.2, 185.7, 197.1, 2131.9
CO/1/4 ML Pt – in subsurface	-299.5107	57.0, 62.0, 107.0, 153.8, 201.2, 2133.3
CO/1/4 ML Pt – ontop surface	-301.2411	61.0, 72.1, 493.8, 494.7, 516.1, 2027.4
2xCO/1/4 ML Pt – ontop surface[1]	-314.7290	45.4, 71.5, 97.6, 120.7, 413.2, 452.8, 457.7, 462.0, 477.1, 526.2, 2034.0, 2103.2
CO/1/9 ML Pt – in subsurface	-493.3761	32.5i, 52.6, 77.6, 148.8, 159.1, 2155.9
CO/1/9 ML Pt – ontop surface	-494.7862	54.8, 97.2, 498.1, 505.7, 521.9, 2014.1
2xCO/1/9 ML Pt – ontop surface[1]	-508.1946	83.7, 88.6, 94.4, 112.7, 408.9, 448.5, 458.5, 461.2, 481.1, 527.4, 2049.8, 2100.2
Pt(111) 1/4 ML CO	-58.1488	38.9, 39.5, 371.3, 371.3, 464.3, 2060.0
Pt(111) 1/2 ML CO	-71.6530	72.8, 75.5, 174.3, 177.0, 326.0, 327.4, 334.5, 394.6, 394.8, 443.7, 1719.2, 2078.4
Pt(211) 1/2 ML CO	-80.0015	194.1, 210.2, 386.8, 398.9, 483.2, 2046.3
Pt(211) 1 ML CO	-93.4993	157.2, 192.5, 218.4, 243.2, 372.9,

		393.3, 402.6, 421.4, 464.2, 478.7, 1995.2, 2055.8

Supplementary References

1 Wang, Y. & Wöll, C. IR spectroscopic investigations of chemical and photochemical reactions on metal oxides: bridging the materials gap. *Chem. Soc. Rev.* **46**, 1875-1932, doi:10.1039/c6cs00914j (2017).

2 Yang, C. & Wöll, C. Infrared Reflection-Absorption Spectroscopy (IRRAS) applied to oxides: Ceria as a case study. *Surface Science* **749**, 122550 (2024).

3 Yang, C., Idriss, H., Wang, Y. & Wöll, C. Surface structure and chemistry of CeO_2 powder catalysts determined by surface-ligand infrared spectroscopy (SLIR). *Acc. Chem. Res.* **57**, 3316-3326 (2024).

4 W. Wan *et al.* Highly stable and reactive platinum single atoms on oxygen plasma-functionalized CeO_2 surfaces: nanostructuring and peroxo effects. *Angew. Chem. Int. Ed.* **61**, e202112640 (2022).

5 H..Brunner, U..Mayer & H..Hoffmann. External reflection infrared spectroscopy of anisotropic adsorbate layers on dielectric substrates. *Appl. Spectrosc.* **51**, 209-217 (1997).

6 J. Kattner & H. Hoffmann. External reflection spectroscopy of thin films on dielectric substrates. *Handbook of vibrational spectroscopy* (2006).

7 Kresse, G. & Furthmüller, J. Efficiency of ab-initio total energy calculations for metals and semiconductors using a plane-wave basis set. *Computational materials science* **6**, 15-50 (1996).

8 Kresse, G. & Furthmüller, J. Efficient iterative schemes for ab initio total-energy calculations using a plane-wave basis set. *Physical review B* **54**, 11169 (1996).

9 Blöchl, P. E. Projector augmented-wave method. *Physical review B* **50**, 17953 (1994).

10 Kresse, G. & Joubert, D. From ultrasoft pseudopotentials to the projector augmented-wave method. *Physical review b* **59**, 1758 (1999).

11 Mortensen, J. J. *et al.* Bayesian error estimation in density-functional theory. *Physical review letters* **95**, 216401 (2005).

12 Wellendorff, J. *et al.* Density functionals for surface science: Exchange-correlation model development with Bayesian error estimation. *Physical Review B—Condensed Matter and Materials Physics* **85**, 235149 (2012).

13 Dudarev, S. L., Botton, G. A., Savrasov, S. Y., Humphreys, C. & Sutton, A. P. Electron-energy-loss spectra and the structural stability of nickel oxide: An LSDA+ U study. *Physical Review B* **57**, 1505 (1998).

14 Monkhorst, H. J. & Pack, J. D. Special points for Brillouin-zone integrations. *Physical review B* **13**, 5188 (1976).

Coordinates of all DFT structures

CO(g):
C O
1.0000000000000000
15.0000000000000000 0.0000000000000000 0.0000000000000000
0.0000000000000000 15.0000000000000000 0.0000000000000000
0.0000000000000000 0.0000000000000000 15.0000000000000000
1 1
Cartesian
5.1064678246669599 1.3931602979935502 5.8406657829783937
4.0763349310001926 0.9011982722069156 5.8837604127434693

CeO_2(111) (2x2):
Ce O
1.0000000000000000
7.7546043670124716 0.0000000000000000 0.0000000000000000
-3.8773021835062353 6.7156843781305469 0.0000000000000000
0.0000000000000000 0.0000000000000000 27.0000000000000000
16 32
Selective dynamics
Cartesian
1.9217702320559096 5.6222694619477540 7.0659203192161950 F F F
-1.9555319519519001 5.6222694619477540 7.0659203192161950 F F F
-0.0168808599479957 2.2644272724481049 7.0659203192161950 F F F
3.8604213235582128 2.2644272727377048 7.0659203196257874 F F F
1.9217212382412581 1.1455069132818880 10.2583756106990496 T T T
5.7990510093381946 1.1455736775748611 10.2583532607046806 T T T
3.8602709006605322 4.5033444152332622 10.2589299686896638 T T T
-0.0169890755940667 4.5034407733907225 10.2583952428969312 T T T
5.7994362478781740 3.3836327806279360 13.4930991541119951 T T T
1.9216882468393386 3.3834938013768907 13.4930349609180045 T T T
3.8607713278915443 0.0256877952468467 13.4933239101887210 T T T
7.7380327976678887 0.0258141922003673 13.4930470285625503 T T T
1.9216545595235308 5.6220351110791036 16.7009060072740567 T T T
-1.9554584463178712 5.6222330208987445 16.7009540845854687 T T T
-0.0170337977700232 2.2644742175320935 16.7009583279648126 T T T
3.8601646055810841 2.2643858801696131 16.7008459324151772 T T T
7.7377235070644756 0.0258658133608400 6.2744693261820927 F F F
3.8604213235582678 0.0258658130712402 6.2744693257725004 F F F
1.9217702315543632 3.3837080025708892 6.2744693257725004 F F F
5.7990724155621720 3.3837080025708892 6.2744693257725004 F F F

3.8604213235582128	2.2644272727377048	9.4402733022387011	F	F	F
-0.0168808599479680	4.5029887321145221	7.8573713135962961	F	F	F
-0.0168808599479957	2.2644272724481049	9.4402733018293006	F	F	F
3.8604213235582407	4.5029887324041216	7.8573713140056984	F	F	F
-1.9555319519519001	5.6222694619477540	9.4402733018293006	F	F	F
5.7990724155621454	1.1451465429044725	7.8573713140056984	F	F	F
1.9217702320559096	5.6222694619477540	9.4402733018293006	F	F	F
1.9217702315543361	1.1451465429044725	7.8573713140056984	F	F	F
-0.0166039926270170	4.5036179858859260	12.6818183120186490	T	T	T
7.7376490464468528	0.0270372097620820	11.0698697364806407	T	T	T
3.8602238056217280	4.5030062206916881	12.6816966634130921	T	T	T
3.8603013154149743	0.0267522397304249	11.0688263587810720	T	T	T
5.7992850876818229	1.1444932234577969	12.6817047867517037	T	T	T
1.9207861158002344	3.3839267881705966	11.0700849867407527	T	T	T
1.9223045146066911	1.1444818039996749	12.6820249764873534	T	T	T
5.7992173209450621	3.3843491451710932	11.0701212527477804	T	T	T
7.7380993158867124	0.0265986717537923	15.8826004406988588	T	T	T
3.8603861730607743	0.0264928625500685	15.8833492954460951	T	T	T
3.8609196596826170	2.2649274310665719	14.3188930252866342	T	T	T
5.8001894578166953	1.1451901936559625	17.5092352475139776	T	T	T
-0.0171211806433598	2.2642825806079125	14.3168756674704127	T	T	T
1.9204925735220775	3.3833582333157715	15.8823469322774997	T	T	T
-1.9557556114124384	5.6216000445913661	14.3188563134159708	T	T	T
5.7992866409318102	3.3839839408200660	15.8829311751076148	T	T	T
1.9227634555254207	5.6217784275555243	14.3192174115759201	T	T	T
-0.0166098232034000	4.5034769461258408	17.5095376134233121	T	T	T
3.8602910147312408	4.5028121270266865	17.5089194305596934	T	T	T
1.9222195520788503	1.1450193731637313	17.5097554778553430	T	T	T

CeO_2(111) (3x3):

Ce O

1.0000000000000000

11.6319065500000001	0.0000000000000000	0.0000000000000000
-5.8159532750000000	10.0735265700000003	0.0000000000000000
0.0000000000000000	0.0000000000000000	23.0000000000000000

27 54

Selective dynamics

Cartesian

1.9580720828791747	5.6223754382404980	6.0659070853896537	F	F	F
-1.9192301011286896	5.6223754382404980	6.0659070853896537	F	F	F
0.0194209908752854	2.2645332487407948	6.0659070853896537	F	F	F
3.8967231743814659	2.2645332490303942	6.0659070857991964	F	F	F
3.8967231743814654	4.5030947081177120	9.2317110614461324	F	F	F

0.0194209908753260	4.5030947084072404	9.2317110618555116	F	F	F
-3.8578811926309382	8.9802176268713314	6.0659070853896537	F	F	F
0.0194209908752443	8.9802176271609309	6.0659070857991964	F	F	F
-1.9192301006271300	7.8609368976173428	9.2317110614461324	F	F	F
1.9580720823775337	7.8609368976173428	9.2317110614461324	F	F	F
5.8353742658752425	1.1452525162482476	9.2317110614461324	F	F	F
1.9580720823690201	1.1452525165377749	9.2317110618555116	F	F	F
5.8353742658838392	5.6223754382404980	6.0659070853896537	F	F	F
7.7740253578878136	2.2645332487407948	6.0659070853896537	F	F	F
7.7740253578877709	4.5030947084072404	9.2317110618555116	F	F	F
3.8967231743815072	8.9802176268713314	6.0659070853896537	F	F	F
5.8353742663853980	7.8609368976173428	9.2317110614461324	F	F	F
9.7126764493815489	1.1452525165377749	9.2317110618555116	F	F	F
5.8347168034123555	3.3812926679020725	12.4405587023750996	T	T	T
1.9577400563954865	3.3815713291102738	12.4402458836666554	T	T	T
7.7735012494027940	0.0239214097035616	12.4407134874054108	T	T	T
3.8961044715185036	0.0235900935504523	12.4412335320148202	T	T	T
0.0190288385922961	6.7394584472754993	12.4401525511617876	T	T	T
-1.9202889619780681	3.3812293883739741	12.4405514989480608	T	T	T
0.0189920240913712	0.0237602448161563	12.4409196243371198	T	T	T
-3.8582449838468134	6.7394008291959917	12.4406002045984678	T	T	T
3.8971083935735815	6.7398243684829957	12.4403912001421446	T	T	T
0.0194209908752838	6.7416561674944857	5.2744560919458863	F	F	F
1.9580720823776583	3.3838139788635808	5.2744560919458863	F	F	F
-1.9192301006270061	3.3838139788635808	5.2744560919458863	F	F	F
0.0194209908753260	4.5030947084072404	6.8573580797693543	F	F	F
3.8967231743815076	4.5030947086968380	6.8573580801788969	F	F	F
5.8353742663854398	1.1452525191972081	6.8573580801788969	F	F	F
1.9580720823775744	1.1452525191972081	6.8573580801788969	F	F	F
-1.9192301011286896	5.6223754382404980	8.4402600680023685	F	F	F
1.9580720828791747	5.6223754382404980	8.4402600680023685	F	F	F
-3.8578811926309791	6.7416561677840852	5.2744560923554289	F	F	F
3.8967231738714339	0.0259717869941168	5.2744560919458863	F	F	F
0.0194209908667713	0.0259717869941168	5.2744560919458863	F	F	F
1.9580720828791343	7.8609368973277434	6.8573580801788969	F	F	F
-1.9192301011286474	7.8609368973277434	6.8573580801788969	F	F	F
0.0194209908752443	8.9802176271609309	8.4402600684119111	F	F	F
-3.8578811926309382	8.9802176268713314	8.4402600680023685	F	F	F
0.0194209903651710	2.2645332463710330	8.4402600680023685	F	F	F
3.8967231743729531	2.2645332463710330	8.4402600680023685	F	F	F
5.8353742663854398	3.3838139788635808	5.2744560919458863	F	F	F
7.7740253578877709	4.5030947084072404	6.8573580797693543	F	F	F
9.7126764493901021	1.1452525191972081	6.8573580801788969	F	F	F
5.8353742658838392	5.6223754382404980	8.4402600680023685	F	F	F

3.8967231743814659	6.7416561677840852	5.2744560923554289	F	F	F
7.7740253578792169	0.0259717869941168	5.2744560919458863	F	F	F
5.8353742658838801	7.8609368973277434	6.8573580801788969	F	F	F
3.8967231743815072	8.9802176268713314	8.4402600680023685	F	F	F
7.7740253573776155	2.2645332463710330	8.4402600680023685	F	F	F
0.0197949231593930	6.7403909263158353	10.0565416838084598	T	T	T
1.9578508118859188	3.3832750568972405	10.0566056044665384	T	T	T
0.0181673960036339	4.4998779682857810	11.6232172677137484	T	T	T
3.8964350800487604	4.5002616819229413	11.6219220687328288	T	T	T
3.8968341923243042	2.2619880170387123	13.2513593796817943	T	T	T
0.0183312648434784	2.2601002302117448	13.2512382673542177	T	T	T
-1.9200476692062052	5.6188786829763622	13.2502884553768041	T	T	T
1.9579307215160111	5.6184257954968446	13.2496549443186638	T	T	T
3.8969883988847789	0.0249668194763805	10.0581116959717516	T	T	T
1.9573541162594998	1.1431434450213784	11.6225909981001649	T	T	T
5.8345052064375746	1.1428490521446000	11.6236311173744440	T	T	T
1.9571459813119043	7.8590467435286495	11.6222205066008328	T	T	T
-1.9193137490516134	7.8583445985652336	11.6225152504014133	T	T	T
0.0202218183779177	8.9769031455319617	13.2515047860857180	T	T	T
-3.8567850284888912	8.9772642873390556	13.2503715815756919	T	T	T
-3.8573828012087343	6.7407027741068042	10.0575553721058171	T	T	T
-1.9190656255408185	3.3831685684252966	10.0571755396957681	T	T	T
5.8359796228937615	3.3825242290421662	10.0563153617055807	T	T	T
7.7719415696966463	4.5003160318159674	11.6225497683355670	T	T	T
7.7750568308764381	2.2622973465168310	13.2514124779546449	T	T	T
5.8352020766035775	5.6202474477317583	13.2511696446997078	T	T	T
3.8972965701784625	6.7411515737412779	10.0577146880228394	T	T	T
0.0196112519743180	0.0253279087728655	10.0576990835818076	T	T	T
7.7739408816685227	0.0260517246969931	10.0565433207477071	T	T	T
9.7113514435763761	1.1419802687845158	11.6226367861063409	T	T	T
5.8346405644734505	7.8585569170489729	11.6224075110602811	T	T	T
3.8971272121172600	8.9767220633086904	13.2509273759736175	T	T	T

CeO_2(bulk):

Ce O

1.0000000000000000

5.5194364067420656	0.0000000000000000	0.0000000000000000
0.0000000000000000	5.5194364067420656	0.0000000000000000
0.0000000000000000	0.0000000000000000	5.5194364067420656

4 8

Cartesian

0.1383181748855673	0.1383177406701800	0.1383175330769192
0.1382688950375872	2.8981321295234288	2.8981325388318462
2.8981327337788856	0.1382691330993948	2.8981325202236330

```
   2.8981319930274170   2.8981320845384348   0.1382695404928783
   1.5182914990180352   1.5182918246324753   1.5182915488485647
   4.2776174472094795   4.2776176216261161   4.2776175892596555
   4.2780219314033916   4.2780221543941703   1.5181998687254252
   1.5185096816080270   1.5185092401917912   4.2777453630880373
   4.2780219444029672   1.5182000416153461   4.2780219351492548
   1.5185093702128800   4.2777460306117021   1.5185093751164185
   1.5182001651382548   4.2780220675409906   4.2780219574122684
   4.2777454235120418   1.5185093447681923   1.5185095801348243
```

1/4 ML Pt – ontop surface:

```
Ce   O   Pt
  1.0000000000000000
     7.7546043670124716    0.0000000000000000    0.0000000000000000
    -3.8773021835062353    6.7156843781305469    0.0000000000000000
     0.0000000000000000    0.0000000000000000   27.0000000000000000
   16   32    1
Selective dynamics
Cartesian
   1.9386510920039051   5.5964036485869144 11.1658039756468579   F   F   F
  -1.9386510920039044   5.5964036485869144 11.1658039756468579   F   F   F
  -0.0000000000000003   2.2385614590872658 11.1658039756468579   F   F   F
   3.8773021835062083   2.2385614593768648 11.1658039760564520   F   F   F
   1.9459363083027559   1.1205594781419348 14.3578123054714339   T   T   T
   5.8085592818987157   1.1157059654829471 14.3639990102298469   T   T   T
   3.8745594713048952   4.4733304249684069 14.3426646403019475   T   T   T
   0.0050803252453814   4.4809249227120223 14.3605630427213811   T   T   T
   5.7991977538013906   3.3840445785592665 17.5616268877903678   T   T   T
   1.9430823137057549   3.3583395163056871 17.6021760015496369   T   T   T
   0.0024030627182691   6.7095156939686094 17.6424248778768700   T   T   T
  -3.8527838325090125   6.6897310467282800 17.5398404322323529   T   T   T
   2.0224303769787606   5.6264358329411577 20.8338504352986931   T   T   T
  -1.9452941561006600   5.6125860485181960 21.0152753209164729   T   T   T
  -0.1215336162357543   2.1409562465914838 20.6106144713284039   T   T   T
   3.9190283011291203   2.2840697963296628 20.8974039964464424   T   T   T
   0.0000000000000000   0.0000000000000000 10.3743529826127556   F   F   F
   0.0000000000000278   6.7156843778409474 10.3743529822031633   F   F   F
   1.9386510915023587   3.3578421892100492 10.3743529822031633   F   F   F
  -1.9386510915023039   3.3578421892100492 10.3743529822031633   F   F   F
   3.8773021835062083   2.2385614593768648 13.5401569586691721   F   F   F
   0.0000000000000277   4.4771229187536816 11.9572549700267690   F   F   F
  -0.0000000000000003   2.2385614590872658 13.5401569582597716   F   F   F
   3.8773021835062362   4.4771229190432811 11.9572549704361659   F   F   F
```

```
 -1.9386510920039044    5.5964036485869144 13.5401569582597716     F     F     F
   5.8159532755101404    1.1192807295436324 11.9572549704361659     F     F     F
   1.9386510920039051    5.5964036485869144 13.5401569582597716     F     F     F
   1.9386510915023318    1.1192807295436324 11.9572549704361659     F     F     F
   0.0412282474215962    4.4890573010842747 16.8011329036785213     T     T     T
 -3.8651470178654899    6.7146197398108880 15.1650900081910649     T     T     T
   3.8542952422938592    4.4598714571987923 16.7507640016748169     T     T     T
 -0.0137588396362820    6.7060441909600641 15.1663340219964411     T     T     T
   5.7815134334081222    1.0977111205536316 16.7521239554023040     T     T     T
   1.9346152890179258    3.3529674426312894 15.1596431446829314     T     T     T
   1.9665772730201423    1.1401512156623461 16.7978772177136051     T     T     T
 -1.9302127503872830    3.3687275553487956 15.1768830387539708     T     T     T
   0.1345743335361543    0.0457707314297578 19.9869671887426605     T     T     T
   3.9145038640439056    2.2613306436072524 18.4566077368641430     T     T     T
   0.0026222198946184    6.6137990056764417 20.1158270048131058     T     T     T
 -0.0701622649997538    2.2018905442969468 18.3118293891423960     T     T     T
   1.7703455325136190    3.3380151600438457 19.9780894992407312     T     T     T
 -1.9410019437746882    5.5822423038818076 18.3334699806722270     T     T     T
 -1.8091108934922446    3.4510283470271741 20.0990946826305859     T     T     T
   2.0010127677009657    5.6217060060257271 18.4540824791045068     T     T     T
   0.3222466758151231    4.6227476330798583 22.5197179840484196     T     T     T
   3.7689272126644946    4.5867818683007506 21.6839146526727404     T     T     T
   5.9388201719203852    1.0641779682116763 21.6061668455816331     T     T     T
   1.9539361890319042    1.3354281047168404 22.4174734450656956     T     T     T
   1.2040433081628645    3.0067927894431405 22.9705594868779244     T     T     T
```

1/9 ML Pt – ontop surface:

```
Ce    O Pt
  1.0000000000000000
      11.6319065500000001       0.0000000000000000       0.0000000000000000
      -5.8159532750000000      10.0735265700000003       0.0000000000000000
        0.0000000000000000       0.0000000000000000      23.0000000000000000
   27    54     1
Selective dynamics
Cartesian
   1.9595270856053570    5.6471731714590998    6.0302324236038061     F     F     F
 -1.9335275924458148    5.6239751732539958    6.0111558235290232     F     F     F
   0.0230316894060623    2.2604626137889579    6.0439252276884412     F     F     F
   3.8915842737989372    2.2626427996297895    6.0318607273414235     F     F     F
   3.8999651790263905    4.5046566570419309    9.2496546077251924     T     T     T
   0.0294808790954528    4.5119149321382928    9.2604587118878285     T     T     T
 -3.8588896834968365    8.9968229316523498    6.0386270900957797     F     F     F
   0.0222667837608004    8.9898165408205148    6.0356888096381951     F     F     F
```

-1.9404673287824830	7.8654537266600499	9.1928610726395661	T	T	T
1.9469472433062815	7.8567826530501419	9.2196793884749937	T	T	T
5.8304124741093517	1.1404228068226652	9.2178154651857938	T	T	T
1.9633115263751657	1.1745016071236165	9.2586661616799386	T	T	T
5.8517299143160049	5.6265812833925057	6.0111637168520176	F	F	F
7.7778477438508293	2.2670041319620342	6.0363638268592226	F	F	F
7.7765465966903342	4.5185109717304694	9.2670991785977552	T	T	T
3.8977018695963839	8.9916504677560241	6.0475461354538993	F	F	F
5.8663392047770024	7.8703655912192767	9.2017564702959564	T	T	T
9.7217703486132816	1.1474343805459228	9.2719023028960095	T	T	T
5.8533433148333112	3.2670449348754564	12.4515127059043351	T	T	T
1.9962090865740529	3.2765658746420954	12.6382617495875138	T	T	T
7.7694231999501175	0.0298050874940997	12.4525060877565465	T	T	T
3.9008803401571499	0.0268722219363165	12.4251875903223734	T	T	T
-0.1611633589263812	6.8261100448123972	12.4090280619462803	T	T	T
-1.9182586587107475	3.3824566569753776	12.5801553148749221	T	T	T
0.0369883627544983	0.0670167770308659	12.4667640712382450	T	T	T
-3.8220316982316733	6.7876025111524072	12.5181773219397741	T	T	T
3.9858780194096646	6.8891312257744559	12.3161253218531357	T	T	T
0.0310282470683032	6.7569601831226054	5.2195254518657279	F	F	F
1.9579532913533955	3.3828732272953799	5.2377540648781098	F	F	F
-1.9181742786780647	3.3872056123105048	5.2145400155627337	F	F	F
0.0061025847754075	4.5112953469691055	6.8389135653889701	F	F	F
3.9099844754150523	4.5155142934123322	6.8329825299571567	F	F	F
5.8320356254122121	1.1441227385723050	6.8367425546986169	F	F	F
1.9587918737875936	1.1489853589168377	6.8878088634198491	F	F	F
-1.9351838071130116	5.6355686036383066	8.3936781613763127	F	F	F
1.9624656410268861	5.6626883270390058	8.3806118633412794	F	F	F
-3.8591952510973591	6.7358686269372914	5.2024688742972680	F	F	F
3.8806494735611010	0.0411211370428666	5.2180572311227138	F	F	F
0.0330256707228212	0.0408746135633706	5.2252811637893890	F	F	F
1.9610124928363737	7.8935022133098540	6.8585819016550857	F	F	F
-1.9259041970759454	7.8671945089820099	6.8337799181415306	F	F	F
0.0004585189123194	8.9919165764905173	8.4230029433321238	F	F	F
-3.8603709950098848	8.9821415677108689	8.4191865675217770	F	F	F
0.0099997568451160	2.2608132725363390	8.4246754649499724	F	F	F
3.9120245929639053	2.2711234159322089	8.4096099158034434	F	F	F
5.8379713469942240	3.3918301765369319	5.2136566581004322	F	F	F
7.7766281452875408	4.4969992038017681	6.8446591057718527	F	F	F
9.7163734266071291	1.1456874175659979	6.8558581807059662	F	F	F
5.8542582995481496	5.6293224479091162	8.3992569094736069	F	F	F
3.8911482557841781	6.7600959312405653	5.2211621647988764	F	F	F
7.7791970167756661	0.0350365845921194	5.2173951976067556	F	F	F
5.8412585844709604	7.8663971077878454	6.8458083494743045	F	F	F

3.9200093761215502	8.9892966714677840	8.4449103365322884	F	F	F
7.7695391409845902	2.2414528081704566	8.4195861987693164	F	F	F
-0.0133118145527193	6.7593712748320591	10.0398723376784691	T	T	T
1.9598077307108868	3.4170238432880038	10.0332968926746062	T	T	T
0.1679474869348394	4.6902658403282427	11.8659663227982506	T	T	T
3.9614250647800060	4.6455730621597011	11.6802272057703078	T	T	T
4.0671824133944146	2.1124039742200744	13.2674140946791468	T	T	T
0.0109798052562553	2.0666490966350186	13.3556203991222944	T	T	T
-1.9868942850027369	5.8208106723833213	13.3549231394219845	T	T	T
2.5016441211035660	4.6717605597002043	14.8310362835096257	T	T	T
3.8967981049298985	0.0366246346100139	10.0342448161637190	T	T	T
1.9876038806894161	1.1112862223686693	11.6273864093531145	T	T	T
5.9041454170132806	5.7098944108928125	13.8188511628077251	T	T	T
1.9436423128584561	7.6344996150974778	11.7832310955790760	T	T	T
-1.9567977358471977	7.8840088063950811	11.5828395886612583	T	T	T
-0.0078201820106321	8.9847538496340515	13.2357259929380788	T	T	T
-3.8385832235204553	8.9612776957371381	13.2834000055246619	T	T	T
-3.8436377261993786	6.7348220898548901	10.0500745802003539	T	T	T
-1.8851839849421221	3.4602643640787800	10.1002059462444294	T	T	T
5.8235310181390165	3.3776614684820787	10.0588735885107052	T	T	T
7.6288551742999839	4.5705124243519686	11.7294696299174674	T	T	T
7.6537005825038094	2.1443261761798560	13.3151500694452309	T	T	T
5.8744336432530062	1.1137419510959616	11.5564950001633733	T	T	T
3.9068746885703378	6.7964510763677817	10.0086585626209601	T	T	T
0.0310868242391371	0.0215890313915101	10.0761299705829419	T	T	T
1.9608817563232090	9.9963470383895974	10.0921124597887495	T	T	T
9.6705970023537571	1.1193622629867552	11.6013691330110120	T	T	T
5.8796945341300546	7.7990171493120899	11.6408250252022754	T	T	T
3.9038069638774342	8.9783519487672372	13.2088158033238869	T	T	T
4.2609430823731449	5.1905627271041501	14.6929319908032028	T	T	T

1/4 ML Pt – in subsurface:

Ce Pt O

1.0000000000000000

7.8056618206116797	0.0000000000000000	0.0000000000000000
-3.9028309103058301	6.7599014300000002	0.0000000000000000
0.0000000000000000	0.0000000000000000	27.0000000000000000

16 1 32

Selective dynamics

Cartesian

1.9514154554053658	5.6332511918124117	11.1658039756468579	F	F	F
-1.9514154554053498	5.6332511918124117	11.1658039756468579	F	F	F
0.0000000000000027	2.2533004763751769	11.1658039756468579	F	F	F

3.9028309103058154	2.2533004766666829	11.1658039760564520	F	F	F
1.9508447290075894	1.1176826438921645	14.3528250225780170	T	T	T
5.8550422039437660	1.1197709050934661	14.3557736127118645	T	T	T
3.9045377044488960	4.5160286589731458	14.3582021458359677	T	T	T
-0.0020327909231259	4.5132986540147488	14.3272081049158260	T	T	T
-1.8864983021095783	3.3762967944670050	17.5277529816170770	T	T	T
1.8897160642820721	3.3803220729598746	17.4959768428156295	T	T	T
-0.0021996850378683	6.7564796602130626	17.5851700638072117	T	T	T
3.9018490440828213	6.7523965148136718	17.5999853747009496	T	T	T
1.9059354218494988	5.5977224235489773	20.8299770910658850	T	T	T
-1.9194385735605457	5.5804097628776494	20.8741804452947974	T	T	T
0.0257448456997891	2.1311464660545725	20.9140284513327472	T	T	T
3.9350355380207525	1.7011050536859205	22.1960717214094743	T	T	T
3.8871034968758185	3.3067931882672958	19.9167356459210296	T	T	T
0.0000000000000000	0.0000000000000000	10.3743529826127556	F	F	F
0.0000000000000369	6.7599014297084938	10.3743529822031633	F	F	F
1.9514154549005143	3.3799507151457293	10.3743529822031633	F	F	F
-1.9514154549004497	3.3799507151457293	10.3743529822031633	F	F	F
3.9028309103058154	2.2533004766666829	13.5401569586691721	F	F	F
0.0000000000000342	4.5066009533333178	11.9572549700267690	F	F	F
0.0000000000000027	2.2533004763751769	13.5401569582597716	F	F	F
3.9028309103058465	4.5066009536248233	11.9572549704361659	F	F	F
-1.9514154554053498	5.6332511918124117	13.5401569582597716	F	F	F
5.8542463657111998	1.1266502381875880	11.9572549704361659	F	F	F
1.9514154554053658	5.6332511918124117	13.5401569582597716	F	F	F
1.9514154549004841	1.1266502381875880	11.9572549704361659	F	F	F
0.0010439990664866	4.5109831901375443	16.7115425383102902	T	T	T
-0.0000950031943026	0.0016344820450751	15.1324791981362878	T	T	T
3.8979177968837537	4.5625207623925697	16.7653893360198865	T	T	T
3.9032013690818355	0.0008334083575305	15.1649934188723776	T	T	T
5.8731467922396057	1.0991626756846189	16.7533891324449229	T	T	T
1.9522334094088472	3.3790905517552674	15.1432695651100460	T	T	T
1.9339847970835771	1.1009236945962499	16.7467006595281589	T	T	T
5.8523585271352898	3.3785479677735002	15.1546746026562928	T	T	T
3.8470201479702610	6.7529871502580354	19.9851353899445741	T	T	T
3.9032486929331913	2.2618969969834390	18.2003934837913093	T	T	T
3.9831011989015312	0.0839006136640258	20.3642956777210387	T	T	T
0.0070153814209806	2.2416561090111085	18.3320239037408754	T	T	T
1.8836085248461372	3.3206924153968234	19.8955037394703851	T	T	T
-1.8529016955613538	5.6380562753599923	18.4137339108143010	T	T	T
5.9022942587787455	3.2177229196369703	19.9726254255074807	T	T	T
1.8496507657568948	5.6432139824872980	18.4253144996310922	T	T	T
0.1171623207536476	4.3134335513706414	21.7048376822548548	T	T	T
3.8331920082464062	4.2070255321249315	21.7587128841741873	T	T	T

1.7437889754141076 1.0479727342469856 22.0138577746505320 T T T
6.0856572549000756 0.8561739270734349 22.0608639064302920 T T T

1/9 ML Pt – in subsurface:
Ce O Pt
1.0000000000000000
11.6319065500000001 0.0000000000000000 0.0000000000000000
-5.8159532750000000 10.0735265700000003 0.0000000000000000
0.0000000000000000 0.0000000000000000 23.0000000000000000
27 54 1
Selective dynamics
Cartesian
1.9595270856053570 5.6471731714590998 6.0302324236038061 F F F
-1.9335275924458148 5.6239751732539958 6.0111558235290232 F F F
0.0230316894060623 2.2604626137889579 6.0439252276884412 F F F
3.8915842737989372 2.2626427996297895 6.0318607273414235 F F F
3.9604208844065760 4.5229386521314510 9.1695299210147283 T T T
-0.0473211422407731 4.5221786095016618 9.1799537160436895 T T T
-3.8588896834968365 8.9968229316523498 6.0386270900957797 F F F
0.0222667837608004 8.9898165408205148 6.0356888096381951 F F F
-1.9338242978631426 7.8707781655333120 9.2302379975919635 T T T
1.9599041147251288 7.8475500975637829 9.2609567069725074 T T T
5.8318509932768565 1.1460121793116040 9.2380211194635464 T T T
1.9565959339962511 1.1592884063144360 9.2854673047070051 T T T
5.8517299143160049 5.6265812833925057 6.0111637168520176 F F F
7.7778477438508293 2.2670041319620342 6.0363638268592226 F F F
7.7797241098779377 4.4839220278881058 9.2717538440468825 T T T
3.8977018695963839 8.9916504677560241 6.0475461354538993 F F F
5.8523789363349632 7.8666976117007819 9.2469638187296184 T T T
9.7176670095835629 1.1475127969452821 9.2751182888754968 T T T
5.8779030638426475 3.3131206909076334 12.4990820792282591 T T T
1.9905619458388291 2.8038268455335467 13.8258701544958882 T T T
1.9534353565137321 10.0593028604354195 12.4576482692499084 T T T
-1.9101992069010914 10.0709589696865898 12.4756935568320770 T T T
-0.0678780534135806 6.7901562540824560 12.4679906132227245 T T T
9.6868626293896334 3.3230165789632342 12.5857317843030501 T T T
-5.7994918712837391 10.0552588972667518 12.5199784848195250 T T T
-3.8528384507487496 6.7216998266355885 12.4888557007012224 T T T
3.9765407434358324 6.7641620736645152 12.4871463739265636 T T T
0.0310282470683032 6.7569601831226054 5.2195254518657279 F F F
1.9579532913533955 3.3828732272953799 5.2377540648781098 F F F
-1.9181742786780647 3.3872056123105048 5.2145400155627337 F F F
0.0061025847754075 4.5112953469691055 6.8389135653889701 F F F

3.9099844754150523	4.5155142934123322	6.8329825299571567	F	F	F
5.8320356254122121	1.1441227385723050	6.8367425546986169	F	F	F
1.9587918737875936	1.1489853589168377	6.8878088634198491	F	F	F
-1.9351838071130116	5.6355686036383066	8.3936781613763127	F	F	F
1.9624656410268861	5.6626883270390058	8.3806118633412794	F	F	F
-3.8591952510973591	6.7358686269372914	5.2024688742972680	F	F	F
3.8806494735611010	0.0411211370428666	5.2180572311227138	F	F	F
0.0330256707228212	0.0408746135633706	5.2252811637893890	F	F	F
1.9610124928363737	7.8935022133098540	6.8585819016550857	F	F	F
-1.9259041970759454	7.8671945089820099	6.8337799181415306	F	F	F
0.0004585189123194	8.9919165764905173	8.4230029433321238	F	F	F
-3.8603709950098848	8.9821415677108689	8.4191865675217770	F	F	F
0.0099997568451160	2.2608132725363390	8.4246754649499724	F	F	F
3.9120245929639053	2.2711234159322089	8.4096099158034434	F	F	F
5.8379713469942240	3.3918301765369319	5.2136566581004322	F	F	F
7.7766281452875408	4.4969992038017681	6.8446591057718527	F	F	F
9.7163734266071291	1.1456874175659979	6.8558581807059662	F	F	F
5.8542582995481496	5.6293224479091162	8.3992569094736069	F	F	F
3.8911482557841781	6.7600959312405653	5.2211621647988764	F	F	F
7.7791970167756661	0.0350365845921194	5.2173951976067556	F	F	F
5.8412585844709604	7.8663971077878454	6.8458083494743045	F	F	F
3.9200093761215502	8.9892966714677840	8.4449103365322884	F	F	F
7.7695391409845902	2.2414528081704566	8.4195861987693164	F	F	F
-0.0047022222270998	6.7581617739175384	10.0510706347837697	T	T	T
1.9594581075823527	3.4304014663415292	9.9205769049118206	T	T	T
-0.0289566293371939	4.5243484122573374	11.6412795922671961	T	T	T
3.9787033900234965	4.4710548174516038	11.6402415349721977	T	T	T
4.1390463502186279	2.0393246678792103	13.6517452932068508	T	T	T
-0.0899488547747582	1.9172731920056660	13.7449849022735719	T	T	T
-1.9216058031220760	5.7751546414249324	13.3429390479028314	T	T	T
1.9748964046701043	5.4731851530497915	13.4233949707172187	T	T	T
3.8288525281878716	0.0546521920458458	10.0833127893798604	T	T	T
1.9843545740626980	1.2359059956349880	11.9458287621920363	T	T	T
5.8213812312579787	5.6253748511355086	13.3325618751322565	T	T	T
1.9567128277459984	7.8179754362147964	11.6791995177715719	T	T	T
-1.9276137649900318	7.8774986428523146	11.6096369300185600	T	T	T
0.0092252966808557	8.9482676820489608	13.2843339726846921	T	T	T
-3.8490142178421376	8.9282269398649099	13.3269423630803203	T	T	T
-3.8532462840083839	6.7284241019553965	10.0715571938794000	T	T	T
-1.9383755130662403	3.3805293762294948	10.0345970818856376	T	T	T
5.8566871387971187	3.3752780087132837	10.0517425502693598	T	T	T
7.7285966240969231	4.4896527586752217	11.6914630393075427	T	T	T
7.6516046239925535	2.1411910829273917	13.3237515304191732	T	T	T
5.8038856599210389	1.1258084785728566	11.6050265751398101	T	T	T

3.9241128063368698 6.7420117404815558 10.0670081928894035 T T T
0.0978301163755256 0.0466941220756091 10.1089545779217769 T T T
1.9496989053355014 10.0735147926581288 10.0826828133385433 T T T
9.7230717754070639 1.0665772201372428 11.6500951820593173 T T T
5.8454675694183971 7.8408670783788734 11.6469206720444234 T T T
3.8982831693088569 8.8987784460513026 13.3150533173482089 T T T
1.9700422270519282 4.5235878536150746 11.6146517581613864 T T T

1/9 ML Pt – substituted:

Ce O Pt
1.0000000000000000
11.6319065500000001 0.0000000000000000 0.0000000000000000
-5.8159532750000000 10.0735265700000003 0.0000000000000000
0.0000000000000000 0.0000000000000000 23.0000000000000000
26 52 1
Selective dynamics
Cartesian
1.9595270856053568 5.6471731714590998 6.0302324236038061 F F F
-1.9335275924458148 5.6239751732539958 6.0111558235290232 F F F
0.0230316894060623 2.2604626137889579 6.0439252276884412 F F F
3.8915842737989372 2.2626427996297895 6.0318607273414235 F F F
3.9353122384352552 4.5270828614673295 9.1879792453204878 T T T
-0.0150218615761965 4.5143805753492474 9.1879250685997178 T T T
-3.8588896834968365 8.9968229316523498 6.0386270900957797 F F F
0.0222667837608004 8.9898165408205148 6.0356888096381951 F F F
-1.9297993094703472 7.8444404299664292 9.1914875310386552 T T T
1.9562393337341910 7.8642297974458319 9.2657756083817837 T T T
5.8242854552209815 1.1612851095587995 9.2629690626950048 T T T
1.9688646450161054 1.1333498833982780 9.3114682924055892 T T T
5.8517299143160049 5.6265812833925057 6.0111637168520176 F F F
7.7778477438508293 2.2670041319620342 6.0363638268592226 F F F
7.7684320865522967 4.5067785462020815 9.2260489541092543 T T T
3.8977018695963843 8.9916504677560241 6.0475461354538993 F F F
5.8489199192520855 7.8646812532032335 9.2293020349759072 T T T
9.7256059888685105 1.1580516563356633 9.2921154040762204 T T T
5.9229516853503270 3.4478375761972817 12.5049458581019408 T T T
1.9541435820112658 10.1128935452219793 12.5061406045933321 T T T
-1.9645762214789055 9.9261880755747427 12.3620440011571482 T T T
-0.0285073000976822 6.7948029028430330 12.4775659321438042 T T T
9.5843339481729206 3.4324118973144637 12.4893921306936519 T T T
-5.7354289927474156 9.9872718959120697 12.4509313467758513 T T T
-3.8615756824236320 6.7431495182012444 12.4993068739261250 T T T
3.9340024227183528 6.8286501862988160 12.4973197992000813 T T T
0.0310282470683031 6.7569601831226054 5.2195254518657279 F F F

1.9579532913533955	3.3828732272953799	5.2377540648781098	F	F	F
-1.9181742786780649	3.3872056123105048	5.2145400155627337	F	F	F
0.0061025847754075	4.5112953469691055	6.8389135653889701	F	F	F
3.9099844754150523	4.5155142934123322	6.8329825299571567	F	F	F
5.8320356254122121	1.1441227385723050	6.8367425546986169	F	F	F
1.9587918737875938	1.1489853589168377	6.8878088634198491	F	F	F
-1.9351838071130116	5.6355686036383066	8.3936781613763127	F	F	F
1.9624656410268859	5.6626883270390058	8.3806118633412794	F	F	F
-3.8591952510973591	6.7358686269372914	5.2024688742972680	F	F	F
3.8806494735611010	0.0411211370428666	5.2180572311227138	F	F	F
0.0330256707228212	0.0408746135633706	5.2252811637893890	F	F	F
1.9610124928363735	7.8935022133098540	6.8585819016550857	F	F	F
-1.9259041970759454	7.8671945089820099	6.8337799181415306	F	F	F
0.0004585189123192	8.9919165764905173	8.4230029433321238	F	F	F
-3.8603709950098848	8.9821415677108689	8.4191865675217770	F	F	F
0.0099997568451160	2.2608132725363390	8.4246754649499724	F	F	F
3.9120245929639053	2.2711234159322089	8.4096099158034434	F	F	F
5.8379713469942240	3.3918301765369319	5.2136566581004322	F	F	F
7.7766281452875408	4.4969992038017681	6.8446591057718527	F	F	F
9.7163734266071291	1.1456874175659979	6.8558581807059662	F	F	F
5.8542582995481496	5.6293224479091162	8.3992569094736069	F	F	F
3.8911482557841781	6.7600959312405653	5.2211621647988764	F	F	F
7.7791970167756661	0.0350365845921194	5.2173951976067556	F	F	F
5.8412585844709604	7.8663971077878454	6.8458083494743045	F	F	F
3.9200093761215498	8.9892966714677840	8.4449103365322884	F	F	F
7.7695391409845902	2.2414528081704566	8.4195861987693164	F	F	F
0.0097128834694593	6.6944820476222935	10.0582828795236399	T	T	T
1.9615795930676938	3.4026635286665852	10.0710713721970446	T	T	T
0.0065680230347549	4.4081363944186123	11.7501000636544664	T	T	T
3.9009223229483840	4.4569076376159069	11.7423207928470070	T	T	T
-1.9130669058743937	5.7703754193140462	13.3236092688628762	T	T	T
1.9436836607311119	5.6686915115844085	13.3730418450898618	T	T	T
3.9027032003183977	-0.0091038030894978	10.0056223795935235	T	T	T
2.1001747809086986	1.0383251597633232	11.7329434789330040	T	T	T
5.8312332930849813	5.7767079887867645	13.3517114245801665	T	T	T
1.9376000987477526	7.8841573865848504	11.6178050962061743	T	T	T
-1.9162884537766596	7.8413621803052322	11.5555686608510797	T	T	T
-0.0185716673714371	8.9100576617775538	13.2937155390675645	T	T	T
-3.7385691178114926	8.8164170400786794	13.3684412389868932	T	T	T
-3.8606348440218090	6.7313561181984483	10.0392152833528510	T	T	T
-1.9243121930178373	3.4231979308666101	10.0247255008779064	T	T	T
5.8334139635109361	3.4095995597301236	10.0338371552726269	T	T	T
7.7591491095971659	4.5276271643230190	11.6477259667451492	T	T	T
7.7500540513748142	2.1053438119188881	13.3956041743642462	T	T	T

5.6476822011659964 1.1620990234804560 11.7520450666205658 T T T
3.9057780105059070 6.7175031109363204 10.0645609006040466 T T T
0.0546570481851753 0.0354500745183127 10.0359736971155513 T T T
1.9325488923929914 10.1523011212359524 10.1254918006957109 T T T
9.7842085607880058 1.1710378006489790 11.7110152224395048 T T T
5.8384783510735483 7.8508469864345329 11.6054404341897026 T T T
3.8010843843633357 8.8899475721263332 13.3458727813128544 T T T
1.9540889591872461 4.4183675996724565 11.8317037856799079 T T T

1/9 ML Pt – in TRANSITION STATE:
Ce O Pt
1.0000000000000000
11.6319065500000001 0.0000000000000000 0.0000000000000000
-5.8159532750000000 10.0735265700000003 0.0000000000000000
0.0000000000000000 0.0000000000000000 23.0000000000000000
27 54 1
Selective dynamics
Cartesian
1.9595270856053570 5.6471731714590998 6.0302324236038061 F F F
-1.9335275924458148 5.6239751732539958 6.0111558235290232 F F F
0.0230316894060623 2.2604626137889579 6.0439252276884412 F F F
3.8915842737989372 2.2626427996297895 6.0318607273414235 F F F
-3.8588896834968365 8.9968229316523498 6.0386270900957797 F F F
0.0222667837608004 8.9898165408205148 6.0356888096381951 F F F
5.8517299143160049 5.6265812833925057 6.0111637168520176 F F F
7.7778477438508293 2.2670041319620342 6.0363638268592226 F F F
3.8977018695963839 8.9916504677560241 6.0475461354538993 F F F
3.8463869647796147 4.5077299857936453 9.1603855196506885 T T T
0.0456340137104134 4.5017408051777146 9.3299944364473433 T T T
-1.9258181928780749 7.8465074930372936 9.1977658966369447 T T T
1.9477638315327921 7.8767669034897816 9.2630226022100626 T T T
5.8105159636909454 1.1587735800072467 9.2433292055269227 T T T
1.9478054526108433 1.2082259797184969 9.2604294838043639 T T T
7.7755547271352299 4.5317895144362286 9.2665314118337303 T T T
5.8823065549915858 7.8775154287374480 9.1535809111795263 T T T
9.7084738591392039 1.1704584224047505 9.2627494425629244 T T T
5.8901688144572404 3.3682562878534261 12.5158116635076766 T T T
1.9127817501029116 2.7150836981539439 13.5191810284088234 T T T
7.7192210403293586 0.0547463254374023 12.4614624598870805 T T T
-1.8883311283391118 10.0566455755537500 12.4182063719076385 T T T
-0.1480354866088196 6.7922495621036028 12.4637001112348269 T T T
-1.9164740539519891 3.4120769403972608 12.5428209273333113 T T T
0.0141630039244519 0.0666996371637629 12.4150130183068015 T T T
-3.8179227585537046 6.7461614702049078 12.4864137484013327 T T T

4.1785816650839003	7.0697154761731849	12.3153712143766541	T	T	T
0.0310282470683032	6.7569601831226054	5.2195254518657279	F	F	F
1.9579532913533955	3.3828732272953799	5.2377540648781098	F	F	F
-1.9181742786780647	3.3872056123105048	5.2145400155627337	F	F	F
0.0061025847754075	4.5112953469691055	6.8389135653889701	F	F	F
3.9099844754150523	4.5155142934123322	6.8329825299571567	F	F	F
5.8320356254122121	1.1441227385723050	6.8367425546986169	F	F	F
1.9587918737875936	1.1489853589168377	6.8878088634198491	F	F	F
-1.9351838071130116	5.6355686036383066	8.3936781613763127	F	F	F
1.9624656410268861	5.6626883270390058	8.3806118633412794	F	F	F
-3.8591952510973591	6.7358686269372914	5.2024688742972680	F	F	F
3.8806494735611010	0.0411211370428666	5.2180572311227138	F	F	F
0.0330256707228212	0.0408746135633706	5.2252811637893890	F	F	F
1.9610124928363737	7.8935022133098540	6.8585819016550857	F	F	F
-1.9259041970759454	7.8671945089820099	6.8337799181415306	F	F	F
0.0004585189123194	8.9919165764905173	8.4230029433321238	F	F	F
-3.8603709950098848	8.9821415677108689	8.4191865675217770	F	F	F
0.0099997568451160	2.2608132725363390	8.4246754649499724	F	F	F
3.9120245929639053	2.2711234159322089	8.4096099158034434	F	F	F
5.8379713469942240	3.3918301765369319	5.2136566581004322	F	F	F
7.7766281452875408	4.4969992038017681	6.8446591057718527	F	F	F
9.7163734266071291	1.1456874175659979	6.8558581807059662	F	F	F
5.8542582995481496	5.6293224479091162	8.3992569094736069	F	F	F
3.8911482557841781	6.7600959312405653	5.2211621647988764	F	F	F
7.7791970167756661	0.0350365845921194	5.2173951976067556	F	F	F
5.8412585844709604	7.8663971077878454	6.8458083494743045	F	F	F
3.9200093761215502	8.9892966714677840	8.4449103365322884	F	F	F
7.7189514134569324	2.2929299780228125	8.4751248200347540	T	T	T
0.0234178712068447	6.7596375579543029	10.0593023078070853	T	T	T
1.9988972542155363	3.4318616197012206	9.9008376749901181	T	T	T
0.2605791077739487	4.4590182045542868	12.1143123491240807	T	T	T
3.4871536471037201	4.7085556541561164	12.2554572451173271	T	T	T
4.1104341657178152	2.0600654406672949	13.4668940663377406	T	T	T
-0.1859980526238891	1.9343203551427792	13.6458617189648006	T	T	T
-2.0071898663127912	5.7954514752000934	13.3568763675239328	T	T	T
1.0720258996422340	6.2459801915885445	14.6732024174539717	T	T	T
3.8715293914878819	0.0457374163470630	10.0570105293845682	T	T	T
1.9456070601339359	1.2228584045672426	11.7699320352151382	T	T	T
5.8931648941924308	5.7271834703271551	13.1971629307466234	T	T	T
1.9914400476848924	7.8675229340712889	11.7149244545009950	T	T	T
-1.9417117580457028	7.8735129375765016	11.5777709375966342	T	T	T
-0.0341047207864644	9.0359558356672842	13.2432912038097736	T	T	T
-3.8219871841543394	9.0119346095935686	13.3069236192289821	T	T	T
-3.8286943940538563	6.7249137926770555	10.0357666293268544	T	T	T

-1.8796567274518048 3.4184094827642459 10.0509618592193508 T T T
5.6463421379380545 3.4872319520232575 10.1970323137190544 T T T
7.7320767747011292 4.4923227768578782 11.6248527821377312 T T T
7.5835979068979160 2.2254119049871437 13.3455490274178459 T T T
5.8521763644204867 1.1590514878157030 11.5743533243144228 T T T
3.9453626717844612 6.7328691642389096 10.0672898446614152 T T T
0.0314076869519042 0.0834173475976127 10.0435307876960245 T T T
1.9331219297912758 10.0733797078172689 10.0672748694819276 T T T
9.6524293347730143 1.1146476928442450 11.6123294464863545 T T T
6.0008646898724702 7.9774731806651014 11.5188741478497274 T T T
4.0062365591900635 9.0319417519858884 13.2792534147002392 T T T
2.3092027421615171 5.4705234186930767 13.5732815784860676 T T T

Pt(111):
Pt
1.0000000000000000
5.6481144096769151 0.0000000000000000 0.0000000000000000
2.8240572048384576 4.8914105622611554 0.0000000000000000
0.0000000000000000 0.0000000000000000 36.9174991562847339
16
Selective dynamics
Cartesian
0.0000000000000000 0.0000000000000000 14.9999999999999236 F F F
2.8240572048384576 0.0000000000000000 14.9999999999999236 F F F
1.4120286024192288 2.4457052811305777 14.9999999999999236 F F F
4.2360858072576866 2.4457052811305777 14.9999999999999236 F F F
1.4120286024192088 0.8152350937101810 17.3058330520947976 F F F
4.2360858072576661 0.8152350937101810 17.3058330520947976 F F F
2.8240572048384376 3.2609403748407586 17.3058330520947976 F F F
5.6481144096768947 3.2609403748407586 17.3058330520947976 F F F
5.6486668743342454 1.6295073553918398 19.5880496713790890 T T T
2.8246096254425388 1.6295070000805465 19.5880500749503774 T T T
7.0606953123845324 4.0752138230948010 19.5880520602832107 T T T
4.2366383371081175 4.0752136820251357 19.5880527159091962 T T T
2.8240283803635720 4.8906532545390302 21.9071582442951680 T T T
5.6480868994161142 4.8906535906376574 21.9071597943659633 T T T
1.4120007889725577 2.4449487377960519 21.9071587524979812 T T T
4.2360571903746953 2.4449480189574242 21.9071580256063640 T T T

Pt(211):
Pt
1.0000000000000000

6.9455237383511985 0.0000000000000000 0.0000000000000000
0.0000000000000000 5.6709963851161111 0.0000000000000000
0.0000000000000000 0.0000000000000000 28.0039160461804997
24
Selective dynamics
Cartesian
4.3517892220713801 1.4044484483081197 17.7491464376886938 T T T
4.3528945002248376 4.2398930750264654 17.7489907674514384 T T T
2.0909033662648659 5.6592988257266734 17.1537775728085897 T T T
2.0907017294310375 2.8244177338139309 17.1540435782494036 T T T
6.8203276019021022 1.4067247819388740 16.4750368262828815 T T T
6.8204303489640266 4.2422299762814664 16.4745586677939713 T T T
4.5820602204352419 5.6573630881004338 15.4358712462659025 T T T
4.5818508274521079 2.8219075468607175 15.4357828179143777 T T T
2.2320837753807781 1.4070545672441652 14.7093102684650869 T T T
2.2328333064643520 4.2424523213025198 14.7085101422069098 T T T
6.9278540545469891 5.6606642551233337 13.9002451722566480 T T T
6.9277652924179813 2.8251724121528001 13.9002030650291442 T T T
4.6232981637851047 1.4069427294522800 13.0927723624135961 F F F
4.6232981637851047 4.2424409220103358 13.0927723624135961 F F F
2.3081235843347385 5.6601900182893639 12.2742345400336959 F F F
2.3081235843347385 2.8246918257313078 12.2742345400336959 F F F
6.9384727432355211 1.4069427294522800 11.4556967176535967 F F F
6.9384727432355211 4.2424409220103358 11.4556967176535967 F F F
4.6232981637851047 5.6601900182893639 10.6371588952735028 F F F
4.6232981637851047 2.8246918257313078 10.6371588952735028 F F F
2.3081235843347385 1.4069427294522800 9.8186210728936043 F F F
2.3081235843347385 4.2424409220103358 9.8186210728936043 F F F
6.9384727432355211 5.6601900182893639 9.0000832505135069 F F F
6.9384727432355211 2.8246918257313078 9.0000832505135069 F F F

$CO/CeO_2(111)$ (2x2):
Ce O C
1.0000000000000000
7.7546043670124716 0.0000000000000000 0.0000000000000000
-3.8773021835062353 6.7156843781305469 0.0000000000000000
0.0000000000000000 0.0000000000000000 27.0000000000000000
16 33 1
Selective dynamics
Cartesian
1.9217702320559096 5.6222694619477540 7.0659203192161950 F F F
-1.9555319519519001 5.6222694619477540 7.0659203192161950 F F F
-0.0168808599479957 2.2644272724481049 7.0659203192161950 F F F

3.8604213235582128	2.2644272727377048	7.0659203196257874	F	F	F
1.9216126275702492	1.1453451094718856	10.2580414049770088	T	T	T
5.7994819284352870	1.1453716290377463	10.2579621698888221	T	T	T
3.8605180606385723	4.5032123150196641	10.2605083952645799	T	T	T
-0.0168501126642711	4.5029904121511111	10.2581035891979777	T	T	T
5.8015040165407541	3.3816168516330096	13.4938993541824246	T	T	T
1.9198648326147705	3.3816715501070624	13.4936674966240560	T	T	T
3.8606146764641003	0.0254235287716675	13.4890561374265889	T	T	T
7.7378671393145950	0.0282802349664420	13.4945574619216728	T	T	T
1.9187106097842750	5.6225576702402416	16.6934776177452804	T	T	T
-1.9536596687076997	5.6229262758504035	16.6933593305897858	T	T	T
-0.0166023678403002	2.2673633304073717	16.7265653151960620	T	T	T
3.8596033817406847	2.2605128079058643	16.6941514792723069	T	T	T
7.7377235070644756	0.0258658133608400	6.2744693261820927	F	F	F
3.8604213235582678	0.0258658130712402	6.2744693257725004	F	F	F
1.9217702315543632	3.3837080025708892	6.2744693257725004	F	F	F
5.7990724155621720	3.3837080025708892	6.2744693257725004	F	F	F
3.8604213235582128	2.2644272727377048	9.4402733022387011	F	F	F
-0.0168808599479680	4.5029887321145221	7.8573713135962961	F	F	F
-0.0168808599479957	2.2644272724481049	9.4402733018293006	F	F	F
3.8604213235582407	4.5029887324041216	7.8573713140056984	F	F	F
-1.9555319519519001	5.6222694619477540	9.4402733018293006	F	F	F
5.7990724155621454	1.1451465429044725	7.8573713140056984	F	F	F
1.9217702320559096	5.6222694619477540	9.4402733018293006	F	F	F
1.9217702315543361	1.1451465429044725	7.8573713140056984	F	F	F
-0.0170963070605215	4.5069710073239042	12.6819908692388736	T	T	T
7.7377126589527654	0.0285356768244274	11.0699174506048763	T	T	T
3.8607163038254724	4.5028668482536869	12.6765178712645010	T	T	T
3.8606011142371095	0.0265182520040241	11.0670746174675969	T	T	T
5.7967108770147204	1.1423072838498660	12.6819339680686962	T	T	T
1.9197224339823127	3.3828887601520665	11.0702486344060915	T	T	T
1.9249563310926199	1.1422996116576631	12.6825160344069321	T	T	T
5.8007732668088163	3.3829085255621818	11.0700690114231719	T	T	T
-0.0182880120845518	0.0424771245414345	15.8924318891052181	T	T	T
3.8592450198013375	0.0274055787432625	15.8592607707021145	T	T	T
3.8601106619372452	2.2729765162254716	14.3172316269686419	T	T	T
5.7705526158754417	1.1265869974364286	17.5139302331897326	T	T	T
-0.0169170079660566	2.2653947762507851	14.3114532574012330	T	T	T
1.9104480530913681	3.3751620298353417	15.8924485302363792	T	T	T
-1.9636067608733216	5.6166778767874508	14.3194578296669874	T	T	T
-1.9463620083505542	3.3748239409202361	15.8927252287858920	T	T	T
1.9297669155913453	5.6164081514108402	14.3190388286834462	T	T	T
-0.0167860570233245	4.5291393933990580	17.5053928077009928	T	T	T
3.8608084807698506	4.5048880585594446	17.5091837937353318	T	T	T

1.9466991995860405 1.1278327313633663 17.5118998143560276 T T T
-0.1507923174148025 1.6911884301898619 20.8077280948909902 T T T
-0.0814377930182946 2.0056422220315460 19.7228142625091891 T T T

CO/CeO_2(111) (3x3):
Ce O C
1.0000000000000000
11.6319065500000001 0.0000000000000000 0.0000000000000000
-5.8159532750000000 10.0735265700000003 0.0000000000000000
0.0000000000000000 0.0000000000000000 23.0000000000000000
27 55 1
Selective dynamics
Cartesian
1.9580720828791747 5.6223754382404980 6.0659070853896537 F F F
-1.9192301011286896 5.6223754382404980 6.0659070853896537 F F F
0.0194209908752854 2.2645332487407948 6.0659070853896537 F F F
3.8967231743814659 2.2645332490303942 6.0659070857991964 F F F
3.8967231743814654 4.5030947081177120 9.2317110614461324 F F F
0.0194209908753260 4.5030947084072404 9.2317110618555116 F F F
-3.8578811926309382 8.9802176268713314 6.0659070853896537 F F F
0.0194209908752443 8.9802176271609309 6.0659070857991964 F F F
-1.9192301006271300 7.8609368976173428 9.2317110614461324 F F F
1.9580720823775337 7.8609368976173428 9.2317110614461324 F F F
5.8353742658752425 1.1452525162482476 9.2317110614461324 F F F
1.9580720823690201 1.1452525165377749 9.2317110618555116 F F F
5.8353742658838392 5.6223754382404980 6.0659070853896537 F F F
7.7740253578878136 2.2645332487407948 6.0659070853896537 F F F
7.7740253578877709 4.5030947084072404 9.2317110618555116 F F F
3.8967231743815072 8.9802176268713314 6.0659070853896537 F F F
5.8353742663853980 7.8609368976173428 9.2317110614461324 F F F
9.7126764493815489 1.1452525165377749 9.2317110618555116 F F F
5.8372161880555051 3.3803299879984094 12.4367172637229757 T T T
1.9575894581062785 3.3818991535474132 12.4637039549834583 T T T
7.7735711195413026 0.0239161840282335 12.4351834060426611 T T T
3.8983937227229597 0.0223724300350073 12.4367301482105432 T T T
0.0186396803604834 6.7431044928212227 12.4367290796094139 T T T
-1.9224346166625932 3.3800273937903329 12.4367000146813425 T T T
0.0163275094163174 0.0221043980924731 12.4368766130558015 T T T
-3.8582015630129547 6.7392217424951406 12.4402895465600878 T T T
3.8964181096345349 6.7430145890580775 12.4366838658988428 T T T
0.0194209908752838 6.7416561674944857 5.2744560919458863 F F F
1.9580720823776583 3.3838139788635808 5.2744560919458863 F F F
-1.9192301006270061 3.3838139788635808 5.2744560919458863 F F F

0.0194209908753260	4.5030947084072404	6.8573580797693543	F	F	F
3.8967231743815076	4.5030947086968380	6.8573580801788969	F	F	F
5.8353742663854398	1.1452525191972081	6.8573580801788969	F	F	F
1.9580720823775744	1.1452525191972081	6.8573580801788969	F	F	F
-1.9192301011286896	5.6223754382404980	8.4402600680023685	F	F	F
1.9580720828791747	5.6223754382404980	8.4402600680023685	F	F	F
-3.8578811926309791	6.7416561677840852	5.2744560923554289	F	F	F
3.8967231738714339	0.0259717869941168	5.2744560919458863	F	F	F
0.0194209908667713	0.0259717869941168	5.2744560919458863	F	F	F
1.9580720828791343	7.8609368973277434	6.8573580801788969	F	F	F
-1.9192301011286474	7.8609368973277434	6.8573580801788969	F	F	F
0.0194209908752443	8.9802176271609309	8.4402600684119111	F	F	F
-3.8578811926309382	8.9802176268713314	8.4402600680023685	F	F	F
0.0194209903651710	2.2645332463710330	8.4402600680023685	F	F	F
3.8967231743729531	2.2645332463710330	8.4402600680023685	F	F	F
5.8353742663854398	3.3838139788635808	5.2744560919458863	F	F	F
7.7740253578877709	4.5030947084072404	6.8573580797693543	F	F	F
9.7126764493901021	1.1452525191972081	6.8573580801788969	F	F	F
5.8353742658838392	5.6223754382404980	8.4402600680023685	F	F	F
3.8967231743814659	6.7416561677840852	5.2744560923554289	F	F	F
7.7740253578792169	0.0259717869941168	5.2744560919458863	F	F	F
5.8353742658838801	7.8609368973277434	6.8573580801788969	F	F	F
3.8967231743815072	8.9802176268713314	8.4402600680023685	F	F	F
7.7740253573776155	2.2645332463710330	8.4402600680023685	F	F	F
0.0189924144185150	6.7388376244299506	10.0576928956624414	T	T	T
1.9579924837048701	3.3833319018470576	10.0535707921616986	T	T	T
0.0258844930893374	4.4966132487606520	11.6325808764996594	T	T	T
3.8885113653689189	4.4963541436270544	11.6319251948162119	T	T	T
3.9215264242123267	2.2489053392646832	13.2495518148415812	T	T	T
-0.0070025593273082	2.2462083670441295	13.2490417900432931	T	T	T
-1.9199840836618833	5.6200454339047825	13.2512753474169216	T	T	T
1.9584483351203867	5.6517112996588654	13.2491802650408061	T	T	T
3.8940875138002782	0.0257609037118350	10.0574714174953570	T	T	T
1.9578300269311959	1.1527148860944703	11.6333109624666804	T	T	T
5.8395569013146522	1.1396224256978995	11.6146375480306006	T	T	T
1.9576904174750478	7.8655436884618872	11.6141769155752215	T	T	T
-1.9213341523677578	7.8579389833261555	11.6192854381312856	T	T	T
0.0186517490192930	8.9768803299502675	13.2484361542932305	T	T	T
-3.8573739600765338	8.9767615902215336	13.2506707812544207	T	T	T
-3.8577218624390817	6.7409429922024069	10.0553580748678488	T	T	T
-1.9167522198458362	3.3850009112841537	10.0573139145271000	T	T	T
5.8340023688166536	3.3849504465516098	10.0573293319106014	T	T	T
7.7735811598990550	4.5016868920740691	11.6191998823998901	T	T	T
7.7743112052475336	2.2618295070733931	13.2484365520841365	T	T	T

5.8364320599155324 5.6192905709917742 13.2508846684833088 T T T
3.8976649309614868 6.7387515475158883 10.0572015439227425 T T T
0.0227735869956829 0.0260668495077923 10.0575567414998233 T T T
7.7741611079762283 0.0260237885146548 10.0515525001496187 T T T
9.7064729074830893 1.1389118122354509 11.6143652390055792 T T T
5.8356434084052786 7.8579546288661177 11.6193480736449430 T T T
3.8978552549566681 8.9769037452767328 13.2490948660042420 T T T
1.9516022750849873 3.4134486910867183 16.5894980169946322 T T T
1.9557265626354321 3.3970529347021423 15.4585720982609303 T T T

CO/1/4 ML Pt – in subsurface:
Ce Pt O C
1.0000000000000000
7.8056618206116797 0.0000000000000000 0.0000000000000000
-3.9028309103058301 6.7599014300000002 0.0000000000000000
0.0000000000000000 0.0000000000000000 30.0000000000000000
16 1 33 1
Selective dynamics
Cartesian
1.9514154554053658 5.6332511918124117 8.8658039756469265 F F F
-1.9514154554053498 5.6332511918124117 8.8658039756469265 F F F
0.0000000000000027 2.2533004763751769 8.8658039756469265 F F F
3.9028309103058154 2.2533004766666829 8.8658039760564122 F F F
1.9514818638659770 1.1172972734388917 12.0540199266154957 T T T
5.8544034408302057 1.1193291369039564 12.0572798191970794 T T T
3.9045207637019121 4.5158397872271241 12.0579023408460273 T T T
-0.0017397081432091 4.5135761898236222 12.0288085124433746 T T T
-1.8870161952605264 3.3747096130077989 15.2286299586805285 T T T
1.8924077093099076 3.3784874530020224 15.1967721670371958 T T T
-0.0011735679537583 6.7547148294886314 15.2966002465737461 T T T
3.9027340945559135 6.7514163671419176 15.3005920139281670 T T T
1.8987997477337428 5.6003577539449685 18.5220587825797622 T T T
-1.9149434513165191 5.5798624193240496 18.5694257093810933 T T T
0.0256262636681233 2.1300738084404793 18.6149220864485443 T T T
3.9320967986774136 1.7065638251071120 19.9286735581853236 T T T
3.8882527335240038 3.2997324682435236 17.6319929918190610 T T T
0.0000000000000000 0.0000000000000000 8.0743529826128224 F F F
0.0000000000000369 6.7599014297084938 8.0743529822031235 F F F
1.9514154549005143 3.3799507151457293 8.0743529822031235 F F F
-1.9514154549004497 3.3799507151457293 8.0743529822031235 F F F
3.9028309103058154 2.2533004766666829 11.2401569586690897 F F F
0.0000000000000342 4.5066009533333178 9.6572549700267274 F F F
0.0000000000000027 2.2533004763751769 11.2401569582598189 F F F

3.9028309103058465 4.5066009536248233 9.6572549704362114 F F F
-1.9514154554053498 5.6332511918124117 11.2401569582598189 F F F
5.8542463657111998 1.1266502381875880 9.6572549704362114 F F F
1.9514154554053658 5.6332511918124117 11.2401569582598189 F F F
1.9514154549004841 1.1266502381875880 9.6572549704362114 F F F
0.0023105630790590 4.5104320852846573 14.4198232374122153 T T T
-0.0000460352097041 0.0019650918748026 12.8371650321260216 T T T
3.8983603197339813 4.5565399675673870 14.4628393248343201 T T T
0.0006543927207661 6.7594293264214986 12.8714587678751968 T T T
5.8710616722302831 1.1002767812842800 14.4576892871140927 T T T
1.9508878173682236 3.3787696966236012 12.8410773340070978 T T T
1.9369251471800721 1.1029975058862480 14.4501271086937777 T T T
5.8543751726562121 3.3777388936822592 12.8559198791539586 T T T
3.8470086628744404 6.7539039525235207 17.6900538838571464 T T T
3.9043202882161814 2.2636143678377447 15.9124981645570784 T T T
3.9847052018948448 0.0574064468619989 18.0328652733734174 T T T
0.0079857606374749 2.2391427835854540 16.0344565483602963 T T T
1.8838228027682655 3.3185361706552086 17.5993710960916161 T T T
-1.8623264522208574 5.6341178728109069 16.1103108666603845 T T T
5.9046663435563556 3.2147240395100631 17.6791854342868504 T T T
1.8609918383990753 5.6386260295153114 16.1204995231427262 T T T
0.1188458550834617 4.3073531296818581 19.4216940329208398 T T T
3.8351186712565983 4.2084433157410963 19.4718848313254966 T T T
1.7491055707456025 1.0399973506077631 19.7137828195651110 T T T
6.0746807672734251 0.8503567995756768 19.7631474604139612 T T T
3.3155547236996914 4.5476798768049678 22.8604113264728426 T T T
3.5335396839936637 3.5838658484776822 22.3010460399963506 T T T

CO/1/4 ML Pt – ontop surface:
Ce O Pt C
1.0000000000000000
7.7546043670124716 0.0000000000000000 0.0000000000000000
-3.8773021835062353 6.7156843781305469 0.0000000000000000
0.0000000000000000 0.0000000000000000 27.0000000000000000
16 33 1 1
Selective dynamics
Cartesian
1.9217702320559096 5.6222694619477540 7.0659203192161950 F F F
-1.9555319519519001 5.6222694619477540 7.0659203192161950 F F F
-0.0168808599479957 2.2644272724481049 7.0659203192161950 F F F
3.8604213235582128 2.2644272727377048 7.0659203196257874 F F F
1.9235283504003080 1.1473787588496911 10.2559404338450904 T T T
5.7973369578529113 1.1472541643294127 10.2536989899138273 T T T

3.8605770006447115	4.5015906344545709	10.2565200551669093	T	T	T
-0.0167581159778516	4.5038340417515688	10.2553696436998294	T	T	T
-1.9498098493693989	3.3837207500489010	13.4949813093232631	T	T	T
1.9188562044008390	3.3864872578971048	13.4958971753856130	T	T	T
3.8639281243743211	0.0252729499811256	13.4927086646026577	T	T	T
7.7368801736541251	0.0277615317774947	13.4734493523405519	T	T	T
1.9517008308379604	5.6424013096796726	16.7046151546710249	T	T	T
-1.9844876710037438	5.6431549457683561	16.6762323890796509	T	T	T
-0.0131396712227463	2.2279099783019474	16.7140816722798036	T	T	T
3.8613483724657200	2.2661190625471299	16.7020884609392795	T	T	T
7.7377235070644756	0.0258658133608400	6.2744693261820927	F	F	F
3.8604213235582678	0.0258658130712402	6.2744693257725004	F	F	F
1.9217702315543632	3.3837080025708892	6.2744693257725004	F	F	F
5.7990724155621720	3.3837080025708892	6.2744693257725004	F	F	F
3.8604213235582128	2.2644272727377048	9.4402733022387011	F	F	F
-0.0168808599479680	4.5029887321145221	7.8573713135962961	F	F	F
-0.0168808599479957	2.2644272724481049	9.4402733018293006	F	F	F
3.8604213235582407	4.5029887324041216	7.8573713140056984	F	F	F
-1.9555319519519001	5.6222694619477540	9.4402733018293006	F	F	F
5.7990724155621454	1.1451465429044725	7.8573713140056984	F	F	F
1.9217702320559096	5.6222694619477540	9.4402733018293006	F	F	F
1.9217702315543361	1.1451465429044725	7.8573713140056984	F	F	F
-0.0179208188390430	4.5069261714981366	12.6892058534101366	T	T	T
7.7359182838365390	0.0296384386369052	11.0714543660250850	T	T	T
3.8644948737987632	4.4925677249497724	12.6727049800202938	T	T	T
3.8622037388636561	0.0250755349523937	11.0670765254326469	T	T	T
5.7880408543134072	1.1516923760043529	12.6831856585663143	T	T	T
1.9175086807439674	3.3860447759592009	11.0706771434078757	T	T	T
1.9353475080873792	1.1489515475297607	12.6780550857888112	T	T	T
-1.9511343597375113	3.3841554134366900	11.0682608649652678	T	T	T
7.7223268012509445	0.0407243525760770	15.8738223378344259	T	T	T
3.8548348256110376	2.2778782010094880	14.3508117121539769	T	T	T
0.0024023585322515	6.7142090261661442	15.8644554083096665	T	T	T
-0.0080076498604142	2.2529282368232559	14.3020656372957689	T	T	T
1.8739966425340528	3.4058861507341067	15.9112439451171781	T	T	T
-1.9697549106292471	5.6271610120667885	14.3076366945766722	T	T	T
-1.9079475842587270	3.3869762786303532	15.8863152036778743	T	T	T
1.9395688738472239	5.6184455951166345	14.3046603089819158	T	T	T
-0.0812572521366892	4.5878001880980515	17.6284628268827959	T	T	T
3.8895988083268191	4.5107474990528518	17.4480969748594035	T	T	T
5.7933518040321195	1.1411386420561207	17.4900297356175685	T	T	T
1.9404472927246839	1.1082692681756436	17.4489147501113351	T	T	T
2.0773302651938046	2.8034952711400494	21.7459883749162728	T	T	T
0.6730016647851600	3.9646008199108786	19.3804606385902076	T	T	T

1.5097502879147049 3.2711662940609667 20.8533136802942209 T T T

2xCO/1/4 ML Pt – ontop surface:

Ce O Pt C

1.0000000000000000

7.7546043670124716 0.0000000000000000 0.0000000000000000

-3.8773021835062353 6.7156843781305469 0.0000000000000000

0.0000000000000000 0.0000000000000000 27.0000000000000000

16 34 1 2

Selective dynamics

Cartesian

1.9000468070369410 5.5776624504787673 7.0660285578129702 F F F
-1.9772553769708689 5.5776624504787673 7.0660285578129702 F F F
-0.0386042849669648 2.2198202609791182 7.0660285578129702 F F F
3.8386978985392437 2.2198202612687181 7.0660285582225644 F F F
1.8996998039485686 1.1040486121530697 10.2545940591416542 T T T
5.7786570752972182 1.1062754636876586 10.2651182938931029 T T T
3.8405794345664046 4.4554527863995901 10.2638274610451941 T T T
-0.0396670716283536 4.4548746293692378 10.2617658993371634 T T T
5.7769092294009212 3.3462375410924112 13.5266985333645380 T T T
1.9035804049272347 3.3427313669330969 13.5024446536505209 T T T
-0.0456069413506390 6.6906077580812706 13.4615272496050480 T T T
3.8563781560561314 6.7033310227727680 13.4778411604574089 T T T
1.9132584520627205 5.6694487952776758 16.6126238940390110 T T T
-1.9575965636881001 5.6520719790251475 16.8070940472620123 T T T
-0.0166245643534508 2.1925358546943250 16.7720699845551877 T T T
3.8305213281360975 2.1525513989259819 16.7192288017078852 T T T
3.8386978985392721 6.6969431800223997 6.2745775647788653 F F F
-0.0386042849669370 6.6969431797328003 6.2745775643692729 F F F
1.9000468065353942 3.3391009911019021 6.2745775643692729 F F F
5.7773489905432029 3.3391009911019021 6.2745775643692729 F F F
3.8386978985392437 2.2198202612687181 9.4403815408352809 F F F
-0.0386042849669366 4.4583817206455345 7.8574795521928777 F F F
-0.0386042849669648 2.2198202609791182 9.4403815404258840 F F F
3.8386978985392717 4.4583817209351340 7.8574795526022783 F F F
-1.9772553769708689 5.5776624504787673 9.4403815404258840 F F F
5.7773489905431763 1.1005395314354856 7.8574795526022783 F F F
1.9000468070369410 5.5776624504787673 9.4403815404258840 F F F
1.9000468065353671 1.1005395314354856 7.8574795526022783 F F F
-0.0304566295111739 4.4420565835542130 12.6863821596900781 T T T
3.8455222082229659 6.6903277307990221 11.0758061105524419 T T T
3.8364822517972468 4.4363309686703953 12.6944459952959843 T T T
-0.0445861270729306 6.6925778693940297 11.0677483835709243 T T T

5.7822657822963528 1.1250104970971813 12.6507603116737428 T T T
1.9010006529507513 3.3411334917871081 11.0621917166724071 T T T
1.9006811826637904 1.1233051222312374 12.6752034499701338 T T T
5.7765586817872752 3.3518240365618537 11.0749660236731486 T T T
3.8618303666808282 6.6028942953333347 15.9604340522887682 T T T
3.8692035672989413 2.1726949604177475 14.3420634884929754 T T T
-0.0367086971286406 6.6065789741638916 15.8965928724311780 T T T
-0.0678767961835105 2.2079379540849118 14.3359612387922937 T T T
1.9664818286662005 3.4329420596683096 15.8298857572696008 T T T
-1.9722594982253934 5.5821551511964502 14.2662738494785160 T T T
5.6867285286001863 3.4102422230638152 16.0814757494422196 T T T
1.9114823013329301 5.6380453843750669 14.2496995346424029 T T T
0.4728174609834581 4.4114677178160635 18.0878932568776278 T T T
3.3607574058284424 4.3534162027401031 18.0789446098736164 T T T
5.6755646865788529 1.0210654398268195 17.5772783880858583 T T T
2.0580384611826839 1.1956703651837874 17.6543738628586269 T T T
4.2453810503659355 3.4873697177981757 21.3403977214876264 T T T
-0.3870196959071825 3.6724127672012026 21.4172749453191713 T T T
1.9159093749239196 4.0212719370932408 19.4662764922093956 T T T
3.3430963096321658 3.6868814151992928 20.6514705611890435 T T T
0.5007013673903993 3.7899482242908538 20.7009199433182971 T T T

CO/1/9 ML Pt – in subsurface:
Ce O Pt C
1.0000000000000000
11.6319065500000001 0.0000000000000000 0.0000000000000000
-5.8159532750000000 10.0735265700000003 0.0000000000000000
0.0000000000000000 0.0000000000000000 23.0000000000000000
27 55 1 1
Selective dynamics
Cartesian
1.9595270856053570 5.6471731714590998 6.0302324236038061 F F F
-1.9335275924458148 5.6239751732539958 6.0111558235290232 F F F
0.0230316894060623 2.2604626137889579 6.0439252276884412 F F F
3.8915842737989372 2.2626427996297895 6.0318607273414235 F F F
3.9605145594040048 4.5223879674391316 9.1700391643122146 T T T
-0.0473162523952557 4.5216968146149101 9.1800887137687468 T T T
-3.8588896834968365 8.9968229316523498 6.0386270900957797 F F F
0.0222667837608004 8.9898165408205148 6.0356888096381951 F F F
-1.9342893598077207 7.8709308461616398 9.2295077474432912 T T T
1.9601022130168921 7.8478019808071977 9.2603971914203100 T T T
5.8320066737721099 1.1452220807833013 9.2379553614219123 T T T
1.9570316706187292 1.1574887419385362 9.2915153681378655 T T T

5.8517299143160049	5.6265812833925057	6.0111637168520176	F	F	F
7.7778477438508293	2.2670041319620342	6.0363638268592226	F	F	F
7.7793516270531100	4.4847761574708436	9.2720302559808569	T	T	T
3.8977018695963839	8.9916504677560241	6.0475461354538993	F	F	F
5.8532342948387699	7.8667700998276704	9.2467790606629308	T	T	T
9.7175481212271269	1.1467018754661498	9.2748287615420271	T	T	T
5.8770968917743369	3.3114163393999156	12.4995404035952262	T	T	T
1.9926522747358706	2.8195107089702103	13.8521368445781832	T	T	T
1.9537099932135256	10.0581775960290791	12.4584077394983623	T	T	T
-1.9158583281771064	10.0726856299141794	12.4727944030493134	T	T	T
-0.0688792434082912	6.7910873395091924	12.4650257876196306	T	T	T
9.6880000665838519	3.3212043101971775	12.5824523351554802	T	T	T
-5.7930081070292418	10.0564254442135699	12.5167292833169554	T	T	T
-3.8526877203388015	6.7215540551880251	12.4919122800915048	T	T	T
3.9784052859084973	6.7648600361702140	12.4854919579311314	T	T	T
0.0310282470683032	6.7569601831226054	5.2195254518657279	F	F	F
1.9579532913533955	3.3828732272953799	5.2377540648781098	F	F	F
-1.9181742786780647	3.3872056123105048	5.2145400155627337	F	F	F
0.0061025847754075	4.5112953469691055	6.8389135653889701	F	F	F
3.9099844754150523	4.5155142934123322	6.8329825299571567	F	F	F
5.8320356254122121	1.1441227385723050	6.8367425546986169	F	F	F
1.9587918737875936	1.1489853589168377	6.8878088634198491	F	F	F
-1.9351838071130116	5.6355686036383066	8.3936781613763127	F	F	F
1.9624656410268861	5.6626883270390058	8.3806118633412794	F	F	F
-3.8591952510973591	6.7358686269372914	5.2024688742972680	F	F	F
3.8806494735611010	0.0411211370428666	5.2180572311227138	F	F	F
0.0330256707228212	0.0408746135633706	5.2252811637893890	F	F	F
1.9610124928363737	7.8935022133098540	6.8585819016550857	F	F	F
-1.9259041970759454	7.8671945089820099	6.8337799181415306	F	F	F
0.0004585189123194	8.9919165764905173	8.4230029433321238	F	F	F
-3.8603709950098848	8.9821415677108689	8.4191865675217770	F	F	F
0.0099997568451160	2.2608132725363390	8.4246754649499724	F	F	F
3.9120245929639053	2.2711234159322089	8.4096099158034434	F	F	F
5.8379713469942240	3.3918301765369319	5.2136566581004322	F	F	F
7.7766281452875408	4.4969992038017681	6.8446591057718527	F	F	F
9.7163734266071291	1.1456874175659979	6.8558581807059662	F	F	F
5.8542582995481496	5.6293224479091162	8.3992569094736069	F	F	F
3.8911482557841781	6.7600959312405653	5.2211621647988764	F	F	F
7.7791970167756661	0.0350365845921194	5.2173951976067556	F	F	F
5.8412585844709604	7.8663971077878454	6.8458083494743045	F	F	F
3.9200093761215502	8.9892966714677840	8.4449103365322884	F	F	F
7.7695391409845902	2.2414528081704566	8.4195861987693164	F	F	F
-0.0051658289432850	6.7586439759883108	10.0507056883776329	T	T	T
1.9591364828447302	3.4316789984394136	9.9230538214320063	T	T	T

-0.0290231745702227	4.5246968182712415	11.6416238097936358	T	T	T
3.9787189802559730	4.4709383292664562	11.6411750868071451	T	T	T
4.1358471665993424	2.0430118896295459	13.6516713373340437	T	T	T
-0.0846370681009321	1.9209235510181797	13.7397469396973051	T	T	T
-1.9178185554791558	5.7746597367661234	13.3492189457547568	T	T	T
1.9732051668445023	5.4665957707076096	13.4326481322114333	T	T	T
3.8353425396598162	0.0520444498661991	10.0781625797447205	T	T	T
1.9863302514253727	1.2113716272625203	11.9227520119879102	T	T	T
5.8207164687730861	5.6270681382973020	13.3356437499817488	T	T	T
1.9578667217716026	7.8147298676432335	11.6801521397969239	T	T	T
-1.9294962608048252	7.8767272321437618	11.6106473949417435	T	T	T
0.0104827661846407	8.9464600899770232	13.2844157670524439	T	T	T
-3.8499552299477613	8.9242428937140694	13.3350975688376039	T	T	T
-3.8532932677986000	6.7278887005374859	10.0722609091126287	T	T	T
-1.9387330537586007	3.3809810510228715	10.0348669423757624	T	T	T
5.8570156797983079	3.3758230882453679	10.0519615316220978	T	T	T
7.7287821871427091	4.4900527928081351	11.6941576376281873	T	T	T
7.6517671018827205	2.1402248472409480	13.3259204982545469	T	T	T
5.8036416916938691	1.1264283127331578	11.6061734771148082	T	T	T
3.9257669601585041	6.7421350034613345	10.0670122005527336	T	T	T
0.0928470281903410	0.0452098487535468	10.1050191344623332	T	T	T
1.9491039209388781	10.0711781375748757	10.0832884729684036	T	T	T
9.7228387386335875	1.0655208099878963	11.6503078906367961	T	T	T
5.8474151167486212	7.8415788701302711	11.6478407925579432	T	T	T
3.8982095001493517	8.8969642923474126	13.3168256173776314	T	T	T
0.9802466945110480	4.6684990254152412	17.4943927046587220	T	T	T
1.9697937130033143	4.5209623443248805	11.6195750849737394	T	T	T
1.3540074596306537	4.0745511409141821	16.6085030150250148	T	T	T

CO/1/9 ML Pt – ontop surface:

Ce O Pt C

1.0000000000000000

11.6319065500000001	0.0000000000000000	0.0000000000000000
-5.8159532750000000	10.0735265700000003	0.0000000000000000
0.0000000000000000	0.0000000000000000	23.0000000000000000

27 55 1 1

Selective dynamics

Cartesian

1.9580720828791747	5.6223754382404980	6.0659070853896537	F	F	F
-1.9192301011286896	5.6223754382404980	6.0659070853896537	F	F	F
0.0194209908752854	2.2645332487407948	6.0659070853896537	F	F	F
3.8967231743814659	2.2645332490303942	6.0659070857991964	F	F	F
3.8967231743814654	4.5030947081177120	9.2317110614461324	F	F	F

0.0194209908753260	4.5030947084072404	9.2317110618555116	F	F	F
-3.8578811926309382	8.9802176268713314	6.0659070853896537	F	F	F
0.0194209908752443	8.9802176271609309	6.0659070857991964	F	F	F
-1.9192301006271300	7.8609368976173428	9.2317110614461324	F	F	F
1.9580720823775337	7.8609368976173428	9.2317110614461324	F	F	F
5.8353742658752425	1.1452525162482476	9.2317110614461324	F	F	F
1.9580720823690201	1.1452525165377749	9.2317110618555116	F	F	F
5.8353742658838392	5.6223754382404980	6.0659070853896537	F	F	F
7.7740253578878136	2.2645332487407948	6.0659070853896537	F	F	F
7.7740253578877709	4.5030947084072404	9.2317110618555116	F	F	F
3.8967231743815072	8.9802176268713314	6.0659070853896537	F	F	F
5.8353742663853980	7.8609368976173428	9.2317110614461324	F	F	F
9.7126764493815489	1.1452525165377749	9.2317110618555116	F	F	F
5.8339899360684981	3.3828020742252547	12.4418324647038148	T	T	T
1.9568521942160524	3.3145106866590894	12.4587948361273604	T	T	T
7.7738720940661779	0.0275379418770486	12.4305000412425528	T	T	T
3.8984274471768980	0.0193939090524811	12.4398864617446989	T	T	T
-0.0166545206507704	6.7699996653185224	12.4430377221538819	T	T	T
-1.9175831055278927	3.3794538586489420	12.4446073895537026	T	T	T
0.0175430695445092	0.0180033515060115	12.4380488463691137	T	T	T
-3.8562099057811969	6.7379946750238666	12.4416907243612016	T	T	T
3.9344570797856355	6.7720418675772098	12.4390517505255929	T	T	T
0.0194209908752838	6.7416561674944857	5.2744560919458863	F	F	F
1.9580720823776583	3.3838139788635808	5.2744560919458863	F	F	F
-1.9192301006270061	3.3838139788635808	5.2744560919458863	F	F	F
0.0194209908753260	4.5030947084072404	6.8573580797693543	F	F	F
3.8967231743815076	4.5030947086968380	6.8573580801788969	F	F	F
5.8353742663854398	1.1452525191972081	6.8573580801788969	F	F	F
1.9580720823775744	1.1452525191972081	6.8573580801788969	F	F	F
-1.9192301011286896	5.6223754382404980	8.4402600680023685	F	F	F
1.9580720828791747	5.6223754382404980	8.4402600680023685	F	F	F
-3.8578811926309791	6.7416561677840852	5.2744560923554289	F	F	F
3.8967231738714339	0.0259717869941168	5.2744560919458863	F	F	F
0.0194209908667713	0.0259717869941168	5.2744560919458863	F	F	F
1.9580720828791343	7.8609368973277434	6.8573580801788969	F	F	F
-1.9192301011286474	7.8609368973277434	6.8573580801788969	F	F	F
0.0194209908752443	8.9802176271609309	8.4402600684119111	F	F	F
-3.8578811926309382	8.9802176268713314	8.4402600680023685	F	F	F
0.0194209903651710	2.2645332463710330	8.4402600680023685	F	F	F
3.8967231743729531	2.2645332463710330	8.4402600680023685	F	F	F
5.8353742663854398	3.3838139788635808	5.2744560919458863	F	F	F
7.7740253578877709	4.5030947084072404	6.8573580797693543	F	F	F
9.7126764493901021	1.1452525191972081	6.8573580801788969	F	F	F
5.8353742658838392	5.6223754382404980	8.4402600680023685	F	F	F

3.8967231743814659	6.7416561677840852	5.2744560923554289	F	F	F
7.7740253578792169	0.0259717869941168	5.2744560919458863	F	F	F
5.8353742658838801	7.8609368973277434	6.8573580801788969	F	F	F
3.8967231743815072	8.9802176268713314	8.4402600680023685	F	F	F
7.7740253573776155	2.2645332463710330	8.4402600680023685	F	F	F
0.0094101141130438	6.7385381968337725	10.0580620303882498	T	T	T
1.9585510864049724	3.3711176245671366	10.0528204747757499	T	T	T
0.0687310723139221	4.5164366759933019	11.6685763975504599	T	T	T
3.8505969400906461	4.5181693386509885	11.6643229862041622	T	T	T
3.9143825864564974	2.2265784348945021	13.2239621179531923	T	T	T
-0.0041514098097175	2.2081054803179345	13.2100953611026668	T	T	T
-1.9367685172292426	5.6317541902226269	13.2278971551587787	T	T	T
1.9745019400101866	5.7927231877034968	13.3998703958946237	T	T	T
3.9013959575728809	0.0276258540400327	10.0552972099332720	T	T	T
1.9607736032921024	1.1320746558298407	11.6222106500553650	T	T	T
5.8503930867018239	1.1434590265947422	11.6146054120862203	T	T	T
1.9568999899392399	7.8522621313716279	11.6123735349438917	T	T	T
-1.9225024654391043	7.8650899072451326	11.6153529204997312	T	T	T
0.0115848977448847	8.9773350008482939	13.2426053934829042	T	T	T
-3.8543447062141314	8.9697924890952017	13.2459913062486265	T	T	T
-3.8565438748511300	6.7348912645017700	10.0574272564106462	T	T	T
-1.9015267908856968	3.3944807504951755	10.0702814159886298	T	T	T
5.8191243209023611	3.3944282171068489	10.0677164192125499	T	T	T
7.7715933214490445	4.4881273709643414	11.6182054277705546	T	T	T
7.7771787350470918	2.2661194337065846	13.2487520070740032	T	T	T
5.8461976549164723	5.6296835252042632	13.2362904255568878	T	T	T
3.9074331849699924	6.7411299758946743	10.0565895099183678	T	T	T
0.0172189599517139	0.0270776414782681	10.0550157219363410	T	T	T
7.7725750952135533	0.0217421496472954	10.0494354746672627	T	T	T
9.6917219542381918	1.1393193354253490	11.6099315476170943	T	T	T
5.8412131527633040	7.8631508878176053	11.6148083925807697	T	T	T
3.9044770838869347	8.9762029646216206	13.2409253325421403	T	T	T
1.1999746482351639	3.0951323174075758	17.5140323106890179	T	T	T
1.7086782441468742	4.8412088473680219	15.1479262250661648	T	T	T
1.4091838907747012	3.7992869813228589	16.6183506459154913	T	T	T

2xCO/1/9 ML Pt – ontop surface:

Ce O Pt C

1.0000000000000000

11.6319065500000001	0.0000000000000000	0.0000000000000000
-5.8159532750000000	10.0735265700000003	0.0000000000000000
0.0000000000000000	0.0000000000000000	23.0000000000000000

27 56 1 2

Selective dynamics
Cartesian

1.9580720828791747	5.6223754382404980	6.0659070853896537	F	F	F
-1.9192301011286896	5.6223754382404980	6.0659070853896537	F	F	F
0.0194209908752854	2.2645332487407948	6.0659070853896537	F	F	F
3.8967231743814659	2.2645332490303942	6.0659070857991964	F	F	F
3.8967231743814654	4.5030947081177120	9.2317110614461324	F	F	F
0.0194209908753260	4.5030947084072404	9.2317110618555116	F	F	F
-3.8578811926309382	8.9802176268713314	6.0659070853896537	F	F	F
0.0194209908752443	8.9802176271609309	6.0659070857991964	F	F	F
-1.9192301006271300	7.8609368976173428	9.2317110614461324	F	F	F
1.9580720823775337	7.8609368976173428	9.2317110614461324	F	F	F
5.8353742658752425	1.1452525162482476	9.2317110614461324	F	F	F
1.9580720823690201	1.1452525165377749	9.2317110618555116	F	F	F
5.8353742658838392	5.6223754382404980	6.0659070853896537	F	F	F
7.7740253578878136	2.2645332487407948	6.0659070853896537	F	F	F
7.7740253578877709	4.5030947084072404	9.2317110618555116	F	F	F
3.8967231743815072	8.9802176268713314	6.0659070853896537	F	F	F
5.8353742663853980	7.8609368976173428	9.2317110614461324	F	F	F
9.7126764493815489	1.1452525165377749	9.2317110618555116	F	F	F
5.8053201882626304	3.2893391727483445	12.4469437477544673	T	T	T
1.9819961692426327	3.2900401600051952	12.4444555564539883	T	T	T
7.7942609052410123	0.0440956506553336	12.4585871150574974	T	T	T
3.8964254255694106	0.0386895766162285	12.4526133568737833	T	T	T
-0.0748364018291072	6.8429391792681846	12.4356678991906975	T	T	T
-1.9212142216507886	3.4074986008539265	12.5472967530023283	T	T	T
-0.0014824668507168	0.0443081907309082	12.4593680154304778	T	T	T
-3.7580767281281173	6.8455934898083308	12.4340949970099786	T	T	T
3.8964792605825744	6.7633446259159742	12.4659994368120906	T	T	T
0.0194209908752838	6.7416561674944857	5.2744560919458863	F	F	F
1.9580720823776583	3.3838139788635808	5.2744560919458863	F	F	F
-1.9192301006270061	3.3838139788635808	5.2744560919458863	F	F	F
0.0194209908753260	4.5030947084072404	6.8573580797693543	F	F	F
3.8967231743815076	4.5030947086968380	6.8573580801788969	F	F	F
5.8353742663854398	1.1452525191972081	6.8573580801788969	F	F	F
1.9580720823775744	1.1452525191972081	6.8573580801788969	F	F	F
-1.9192301011286896	5.6223754382404980	8.4402600680023685	F	F	F
1.9580720828791747	5.6223754382404980	8.4402600680023685	F	F	F
-3.8578811926309791	6.7416561677840852	5.2744560923554289	F	F	F
3.8967231738714339	0.0259717869941168	5.2744560919458863	F	F	F
0.0194209908667713	0.0259717869941168	5.2744560919458863	F	F	F
1.9580720828791343	7.8609368973277434	6.8573580801788969	F	F	F
-1.9192301011286474	7.8609368973277434	6.8573580801788969	F	F	F
0.0194209908752443	8.9802176271609309	8.4402600684119111	F	F	F

-3.8578811926309382	8.9802176268713314	8.4402600680023685	F	F	F
0.0194209903651710	2.2645332463710330	8.4402600680023685	F	F	F
3.8967231743729531	2.2645332463710330	8.4402600680023685	F	F	F
5.8353742663854398	3.3838139788635808	5.2744560919458863	F	F	F
7.7740253578877709	4.5030947084072404	6.8573580797693543	F	F	F
9.7126764493901021	1.1452525191972081	6.8573580801788969	F	F	F
5.8353742658838392	5.6223754382404980	8.4402600680023685	F	F	F
3.8967231743814659	6.7416561677840852	5.2744560923554289	F	F	F
7.7740253578792169	0.0259717869941168	5.2744560919458863	F	F	F
5.8353742658838801	7.8609368973277434	6.8573580801788969	F	F	F
3.8967231743815072	8.9802176268713314	8.4402600680023685	F	F	F
7.7740253573776155	2.2645332463710330	8.4402600680023685	F	F	F
-0.0148792226564090	6.7454977745771734	10.0468203506490443	T	T	T
1.9149687248730505	3.3619633670940456	10.0389083029692348	T	T	T
0.2115388193676599	4.6425496686552785	11.7921175044658710	T	T	T
3.8945284510009786	4.4168865741046091	11.5541291919095457	T	T	T
3.8937753345436441	2.1883012110187963	13.2978088704455431	T	T	T
0.1378666815900254	2.1827945326105147	13.2992890526597165	T	T	T
-1.9176942920842339	5.8303294469389817	13.3205755339582197	T	T	T
2.4532323840631034	5.2482677103130229	13.8664817658770190	T	T	T
3.8960582361627196	0.0297935511727733	10.0385007953458558	T	T	T
1.9727514743777603	1.1186058152789662	11.5992144694363475	T	T	T
5.8166106449236521	1.1167192582534893	11.5997034805404340	T	T	T
1.8690642160406750	7.7861537525906304	11.7137242998012852	T	T	T
-1.9173994483620622	7.9053948025895071	11.5952312353566569	T	T	T
-0.0314192700650855	8.9941294194047092	13.3014257764973181	T	T	T
-3.8017395828679015	8.9965235114742388	13.3024019854336331	T	T	T
-3.8217192423019313	6.7462324724677591	10.0478031419743488	T	T	T
-1.9217156461391911	3.4632099345266489	10.0961607941686111	T	T	T
5.8783288776985918	3.3616455312923974	10.0397516197147763	T	T	T
7.5736468178989211	4.6467897891366103	11.7986902981787196	T	T	T
7.6531308057405543	2.1812820158074664	13.2969356687795113	T	T	T
5.3058871897395834	5.2246160867891787	13.8802601420451737	T	T	T
3.8978919483728145	6.7723783402307642	10.0095556417860951	T	T	T
-5.8021546690232473	10.0646506170506065	10.0684887524861519	T	T	T
1.9644665100833203	10.0662671698451440	10.0678069329658015	T	T	T
9.7134381560874417	1.1447342452399110	11.5912453817907135	T	T	T
5.9265583498395111	7.7841608408773846	11.7182618881974463	T	T	T
3.8980578936238821	9.1275971161710530	13.2901937359827116	T	T	T
6.1723558108846728	4.3424064258626736	17.1744110031261812	T	T	T
1.5380986161988961	4.4383302726290577	17.1596785416345980	T	T	T
3.8712446037678707	4.8552441528245502	15.2548252318237161	T	T	T
5.2835608841303552	4.5314002899444610	16.4665237602047583	T	T	T
2.4396606143455615	4.5816823188052203	16.4671272267376381	T	T	T

Pt(111) 1/4 ML CO:

```
Pt    C    O
  1.0000000000000000
        5.6481144096769151         0.0000000000000000         0.0000000000000000
        2.8240572048384576         4.8914105622611554         0.0000000000000000
        0.0000000000000000         0.0000000000000000        36.9174991562847339
   16     1     1
Selective dynamics
Cartesian
    0.0000000000000000    0.0000000000000000  14.9999999999999236     F     F     F
    2.8240572048384576    0.0000000000000000  14.9999999999999236     F     F     F
    1.4120286024192288    2.4457052811305777  14.9999999999999236     F     F     F
    4.2360858072576866    2.4457052811305777  14.9999999999999236     F     F     F
    1.4120286024192088    0.8152350937101810  17.3058330520947976     F     F     F
    4.2360858072576661    0.8152350937101810  17.3058330520947976     F     F     F
    2.8240572048384376    3.2609403748407586  17.3058330520947976     F     F     F
    5.6481144096768947    3.2609403748407586  17.3058330520947976     F     F     F
    5.6283392573074176    1.6415421573928521  19.6066697168790895     T     T     T
    2.8436013647779053    1.6416812233421161  19.6067785191587127     T     T     T
    7.0598917760588593    4.0759634571080765  19.5736962832205705     T     T     T
    4.2358278065363919    4.0530509898683649  19.6066789127334573     T     T     T
    0.0029424987048127    0.0013498433175918  21.8583549324585817     T     T     T
    2.8204278810975603    0.0013040953163115  21.8581537814958011     T     T     T
    1.4115976498228961    2.4414892965770805  21.8585079489105247     T     T     T
    4.2356555871718395    2.4450843491782357  22.0983166341032451     T     T     T
    4.2355036176404406    2.4447548807821655  23.9448981889010000     T     T     T
    4.2353875139980559    2.4443814189640873  25.1120957301021193     T     T     T
```

Pt(111) 1/2 ML CO:

```
Pt    C    O
  1.0000000000000000
        5.6481144096769151         0.0000000000000000         0.0000000000000000
        2.8240572048384576         4.8914105622611554         0.0000000000000000
        0.0000000000000000         0.0000000000000000        36.9174991562847339
   16     2     2
Selective dynamics
Cartesian
    0.0000000000000000    0.0000000000000000  14.9999999999999236     F     F     F
    2.8240572048384576    0.0000000000000000  14.9999999999999236     F     F     F
    1.4120286024192288    2.4457052811305777  14.9999999999999236     F     F     F
    4.2360858072576866    2.4457052811305777  14.9999999999999236     F     F     F
```

1.4120286024192088 0.8152350937101810 17.3058330520947976 F F F
4.2360858072576661 0.8152350937101810 17.3058330520947976 F F F
2.8240572048384376 3.2609403748407586 17.3058330520947976 F F F
5.6481144096768947 3.2609403748407586 17.3058330520947976 F F F
5.6374534947843395 1.6435910130258957 19.6202378613633819 T T T
2.8426438237384253 1.6433055595025834 19.6198859173535141 T T T
7.0639109718491113 4.0801407192806058 19.6292606963696947 T T T
4.2404479079830146 4.0635312166511843 19.6203581973592733 T T T
8.4613601303882930 4.8895965848538747 21.9699252591688179 T T T
5.6728586761452515 4.8897456908819583 21.9709662092859084 T T T
7.0674047334680274 2.4740789237347904 21.9716894794841835 T T T
4.2431962460349562 2.4542595433874257 22.0703775319364368 T T T
1.4199679138378332 0.8238847765623460 23.2773225861986255 T T T
4.2469927574532118 2.4544193225741262 23.9322851274213875 T T T
4.2510298129408657 2.4550288493014647 25.0845807175338784 T T T
1.4189996113807148 0.8222519862514188 24.4868371304712227 T T T

Pt(211) 1/2 ML CO:
Pt C O
1.0000000000000000
6.9455237383511985 0.0000000000000000 0.0000000000000000
0.0000000000000000 5.6709963851161111 0.0000000000000000
0.0000000000000000 0.0000000000000000 29.0039160461804997
24 1 1
Selective dynamics
Cartesian
4.5114709199452978 1.4177492404277796 18.9229690984400065 T T T
4.4181640496515051 4.2532477423147936 18.7428538923757628 T T T
2.1492970362318977 5.6667060094203707 18.1257261332483708 T T T
2.1493008003891929 2.8397908202583348 18.1257265898151267 T T T
6.8948656567973927 1.4177409341933229 17.4348280421626072 T T T
6.8708710524711281 4.2532071720693407 17.4315948413162367 T T T
4.6126218073149019 0.0357424978642424 16.4914554617573437 T T T
4.6126249751608546 2.7997419959507477 16.4914648993810857 T T T
2.2631170442309090 1.4177509636891423 15.6936329544278461 T T T
2.2805565777159367 4.2532475528928000 15.6878374506264944 T T T
6.9321397478159312 5.6686820131843509 14.8944679838593341 T T T
6.9321410634162097 2.8378189424408848 14.8944651038882672 T T T
4.6303491589007821 1.4177490962790278 14.0926891119003059 F F F
4.6303491589007821 4.2532472888370831 14.0926891119003059 F F F
2.3151745794504159 0.0000000000000000 13.2741512895202138 F F F
2.3151745794504159 2.8354981925580556 13.2741512895202138 F F F
0.0000000000000000 1.4177490962790278 12.4556134671401217 F F F

```
   0.0000000000000000  4.2532472888370831 12.4556134671401217   F   F   F
   4.6303491589007821  0.0000000000000000 11.6370756447600314   F   F   F
   4.6303491589007821  2.8354981925580556 11.6370756447600314   F   F   F
   2.3151745794504159  1.4177490962790278 10.8185378223801436   F   F   F
   2.3151745794504159  4.2532472888370831 10.8185378223801436   F   F   F
   0.0000000000000000  0.0000000000000000 10.0000000000000533   F   F   F
   0.0000000000000000  2.8354981925580556 10.0000000000000533   F   F   F
   4.5681148590910006  1.4177504432487205 20.7753925988103525   T   T   T
   4.6253254785977891  1.4177630643641321 21.9432579035838700   T   T   T
```

```
Pt(211) 1 ML CO:
Pt   C   O
 1.0000000000000000
     6.9455237383511985    0.0000000000000000    0.0000000000000000
     0.0000000000000000    5.6709963851161111    0.0000000000000000
     0.0000000000000000    0.0000000000000000   29.0039160461804997
  24    2    2
Selective dynamics
Cartesian
   4.4455681652245946  1.4165037205236870 18.9601623219985633   T   T   T
   4.6550171571413657  4.2532936154363803 18.9680320939755092   T   T   T
   2.1848772288064984  5.6511376134924891 18.0807388752336031   T   T   T
   2.1846776680642224  2.8545058197270001 18.0822252092928757   T   T   T
   6.8791286269608483  1.4168562604514998 17.4429627650791268   T   T   T
   6.9407403416246396  4.2523653375001791 17.3826422538383980   T   T   T
   4.6271702797384933  5.6623975207976525 16.5549827024711824   T   T   T
   4.6270069335956743  2.8364112626601146 16.5554511568305784   T   T   T
   2.2866512450162797  1.4174529124946043 15.6725349743154521   T   T   T
   2.3000389368908034  4.2524753554168200 15.6590542258188261   T   T   T
   6.9206489030850191  0.0037261953331491 14.8968329281900544   T   T   T
   6.9205654600172544  2.8316084484545621 14.8970355269382218   T   T   T
   4.6303491589007821  1.4177490962790278 14.0926891119003059   F   F   F
   4.6303491589007821  4.2532472888370831 14.0926891119003059   F   F   F
   2.3151745794504159  0.0000000000000000 13.2741512895202138   F   F   F
   2.3151745794504159  2.8354981925580556 13.2741512895202138   F   F   F
   0.0000000000000000  1.4177490962790278 12.4556134671401217   F   F   F
   0.0000000000000000  4.2532472888370831 12.4556134671401217   F   F   F
   4.6303491589007821  0.0000000000000000 11.6370756447600314   F   F   F
   4.6303491589007821  2.8354981925580556 11.6370756447600314   F   F   F
   2.3151745794504159  1.4177490962790278 10.8185378223801436   F   F   F
   2.3151745794504159  4.2532472888370831 10.8185378223801436   F   F   F
   0.0000000000000000  0.0000000000000000 10.0000000000000533   F   F   F
   0.0000000000000000  2.8354981925580556 10.0000000000000533   F   F   F
   3.8455486294318524  1.3733791240631246 20.7181740814258717   T   T   T
```

5.1243260691098609	4.2103998158354887	20.7630249626171199	T	T	T
3.4127383455748292	1.3396211587701286	21.7928643965784268	T	T	T
5.4703059142450776	4.1802310848674882	21.8801055355012295	T	T	T
